# Screening and Identification of HBV Epitopes Restricted by Multiple Prevalent HLA-A Allotypes

**DOI:** 10.3389/fimmu.2022.847105

**Published:** 2022-04-07

**Authors:** Yan Ding, Zining Zhou, Xingyu Li, Chen Zhao, Xiaoxiao Jin, Xiaotao Liu, Yandan Wu, Xueyin Mei, Jian Li, Jie Qiu, Chuanlai Shen

**Affiliations:** ^1^ Department of Microbiology and Immunology, Medical School, Southeast University, Nanjing, China; ^2^ Key Laboratory of Developmental Genes and Human Disease, Ministry of Education, School of Life Science and Technology, Southeast University, Nanjing, China; ^3^ Division of Hepatitis, Nanjing Second Hospital, Nanjing Hospital Affiliated to Nanjing University of Chinese Medicine, Nanjing, China

**Keywords:** hepatitis B virus, T cell epitope, HLA-A, ELISPOT, bioinformatics analysis, antigen-specific T cell detection

## Abstract

Although host T cell immune responses to hepatitis B virus (HBV) have been demonstrated to have important influences on the outcome of HBV infection, the development of T cell epitope-based vaccine and T cell therapy and the clinical evaluation of specific T cell function are currently hampered markedly by the lack of validated HBV T cell epitopes covering broad patients. This study aimed to screen T cell epitopes spanning overall HBsAg, HBeAg, HBx and HBpol proteins and presenting by thirteen prevalent human leukocyte antigen (HLA)-A allotypes which gather a total gene frequency of around 95% in China and Northeast Asia populations. 187 epitopes were *in silico* predicted. Of which, 62 epitopes were then functionally validated as real-world HBV T cell epitopes by *ex vivo* IFN-γ ELISPOT assay and *in vitro* co-cultures using peripheral blood mononuclear cells (PBMCs) from HBV infected patients. Furthermore, the HLA-A cross-restrictions of each epitope were identified by peptide competitive binding assay using transfected HMy2.CIR cell lines, and by HLA-A/peptide docking as well as molecular dynamic simulation. Finally, a peptide library containing 105 validated epitopes which cross-binding by 13 prevalent HLA-A allotypes were used in ELISPOT assay to enumerate HBV-specific T cells for 116 patients with HBV infection. The spot forming units (SFUs) was significantly correlated with serum HBsAg level as confirmed by multivariate linear regression analysis. This study functionally validated 62 T cell epitopes from HBV main proteins and elucidated their HLA-A restrictions and provided an alternative ELISPOT assay using validated epitope peptides rather than conventional overlapping peptides for the clinical evaluation of HBV-specific T cell responses.

## 1 Introduction

Hepatitis B virus (HBV) infections are still prevalent across the world. The global patients with chronic infection have exceeded 257 million which results in about 880,000 deaths per year due to liver cirrhosis (LC) or hepatocellular carcinoma (HCC) (1). In China, currently about 70 million people have been infected with HBV, around 20-30 million people have suffered from chronic infections which is the main pathogenic factor for 77% of liver cirrhosis and 84% of HCC (2). Liver inflammation induced by HBV is mainly caused by the host immune responses. Numerous researches have confirmed that HBV-specific T cells not only substantially drive virus clearance (3, 4) and disease progression (5–7), but also significantly influence antiviral efficacy (8, 9) and disease recurrence after therapy discontinuation (10–12). The immune responses mediated by HBV antigen-specific CD8^+^ T cells are particularly critical for host antiviral protections since CD8^+^ cytotoxic T lymphocytes (CTL) are the vital cells to kill virus-infected cells. Human leukocyte antigen (HLA) class I molecules (such as HLA-A, B, and C) expressed by virus-infected liver cells present the HBV epitope peptides to specific CD8^+^ T cells, thus initiate the activation, proliferation and differentiation of CTLs. However, HLA class I molecules are highly polymorphic in the general population. That means HLA class I allotypes are distinctive from individual to individual, and each HLA allotype presents distinctive epitope peptides, thus leading to different strengths of protective or pathogenic immune responses in different individuals against the same pathogen such as HBV (13, 14). Thus far, the validated T cell epitopes in HBV antigens are still very limited, only 205 CD8^+^ T cell epitopes and 79 CD4^+^ T cell epitopes have been defined from HBV proteome by cellular functional experiments, but most are restricted to several common HLA supertypes, such as HLA-A0201, A2402, B0702, DR04, and DR12 molecules, as displayed in the Hepitopes database (15) and other recent reports (6, 16–24). Therefore, the current library of validated HBV T cell epitopes cannot cover a broad population carrying highly polymorphic HLA alleles in the indicated geographical regions, and also can not cover broad antigenic targets recognized by HBV-specific CD8^+^ or CD4^+^ T cell clones. This limitation thus hampered the development of T cell epitope-based therapeutic vaccine and T cell immunotherapy, and also hampered the clinical precise evaluation of HBV-specific cellular immunity.

To achieve more variable CD8^+^ T cell epitopes which cover more dominant HLA-A allotypes and span more HBV proteins, this study dedicated to the mapping of CD8^+^ T cell epitopes derived from overall HBsAg, HBeAg (covering HBcAg), HBx and HBpol proteins, and restricted by a series of high-frequency HLA-A allotypes which gather a total gene frequency of around 95% in Asian population. 187 epitopes restricted by 13 HLA-A allotypes were *in silico* predicted and selected as candidate epitopes. Of these, 62 epitopes were then functionally validated as real-world epitopes by *ex vivo* enzyme-linked immunosorbent spot (ELISPOT) assay and *in vitro* co-cultures, using patients’ peripheral blood mononuclear cells (PBMCs). Furthermore, the peptide competitive binding assay and HLA-A/peptide docking as well as molecular dynamic simulation were used to identify the binding affinity and HLA-A cross-restrictions of the 62 epitopes. Finally, a peptide library containing 105 validated epitopes was used to enumerate the peripheral HBV-specific T cells in IFN-γ ELISPOT assay for 116 patients with HBV infection.

## 2 Materials and Methods

### 2.1 Patient Cohort

Patients with HBV infection were recruited in this study from the Division of Hepatitis or Department of Clinical Laboratory at Nanjing Second Hospital. According to the EASL 2017 Clinical Practice Guidelines on the management of hepatitis B virus infection (11), the CHB patients had clinical, biochemical and virological evidence of chronic hepatitis B infection with HBsAg positiveness for at least 6 months. Three CHB patient groups were enrolled in this study: patients in immune tolerant stage (IT, HBeAg^+^ chronic infection, high viremia but limited liver inflammation), patients in immune active stage (IA, HBeAg^+^ chronic hepatitis, high viremia and high level of alanine aminotransferase), and immune inactive carriers (IC or InA, HBeAg^-^ chronic infection, low or undetectable serum viral load and limited liver inflammation with normal level of alanine aminotransferase). In addition, 29 acute resolved patients (R, low or undetectable serum viral load, HBsAg^-^, and anti-HBcAb^+^), 20 patients with liver cirrhosis and 14 patients with liver cancer were also enrolled here. The exclusion criteria for these subjects were the infection with hepatitis C virus, hepatitis A virus or human immunodeficiency virus, and malignant tumor.

### 2.2 Ethical Approval

The present study was conducted according to the Declaration of Helsinki principles, and the human samples collection and use has been approved by Clinical Ethics Committee of Nanjing Second Hospital (ref: 2018-LY-kt054, 2019-LY-ky011, 2021-LS-ky013). For the validation of putative epitopes using IFN-γ ELISPOT assay, 500 whole blood samples from HBV-infected patients were collected from Department of Clinical Laboratory of Nanjing Second Hospital. In this instance, informed consent was waived because these blood samples were the biological specimens obtained from past clinical diagnosis and treatment, but consent was obtained from Clinical Ethics Committee of Nanjing Second Hospital. For the clinical detection of HBV-specific T cells, 116 participants recruited from Division of Hepatitis of Nanjing Second Hospital gave written, informed consent. 3 to 5 mL of whole blood was taken from each patient, and PBMCs were further isolated by density-gradient centrifugation.

### 2.3 *In Silico* Prediction of CD8^+^ T Cell Epitopes and Peptide Synthesis

T cell epitopes spanning HBsAg, HBeAg (covering HBcAg), HBpol and HBx proteins of HBV and presented by different HLA-A allotypes were *in silico* predicted using six epitope predication tools and eight types of algorithms (SYFPEITHI, BIMAS, SVMHC-SYFPEITHI/MHCPEP, IEDB-ANN/SMM, NetMHC, EPIJEN). For each HLA-A allotype and each protein, one to five 9-mer and 10-mer peptides with the highest score (highest affinity) as predicted by at least two tools were selected as candidate epitopes for further validation. This study mainly focused on adr and adw serotypes and B, C genotypes of HBV, which are common in Chinese population, and also took A and D genotypes into account. The entire amino acid sequences of each protein from different genotypes were obtained from UniProt database and aligned in **Figure S1**.

The peptides were synthesized from China Peptides Co., Ltd with a purity of > 95% as defined by HPLC purification and mass spectrometry and were used in cellular functional experiments. Lyophilized peptides were reconstituted at a stock concentration of 2 mg/mL in DMSO-PBS solution and stored in aliquots at -80°C.

### 2.4 Immunogenicity Validation of Predicted Epitopes by IFN-γ ELISPOT Assay Using Patients’ PBMCs

Peripheral blood samples from patients with HBV infection were collected from Department of Clinical Laboratory at Nanjing Second Hospital and processed into PBMCs. The predicted epitopes restricted by the indicated HLA-A allotypes were grouped into several peptide pools (10 epitopes/pool, 10 μg/epitope/mL), and co-cultured with PBMCs (2×10^5^/well/peptide pool) in 96-well plates coated with anti-human IFN-γ for 20 h followed by IFN-γ ELISPOT assay, according to manufacturer’s instructions (Dakewe Biotech, Shenzhen, China). In parallel, negative control well (PBMCs alone) and positive control well (PBMCs with phytohemagglutinin, PHA, 2.5 μg/mL) were also performed. Notably, in each negative control well, DMSO was supplemented to make its concentration equal to the peptide pool/PBMCs co-culture well. The spot forming units (SFUs) were imaged and enumerated. Positive T cell response was defined according to the criterion as follows: (SFUs in peptide well - SFUs in negative control well) ≧ 5, while SFUs in negative control well was 0-5; or (SFUs in peptide well)/(SFUs in negative control well) ≧ 2, while SFUs in negative control well was > 5. When the peptide pool can significantly stimulate patient’s PBMCs producing IFN-γ, the PBMCs were re-collected from the same individual, then co-cultured with each epitope peptide in the positive peptide pool and followed by IFN-γ ELISPOT assay to identify whether the indicated epitope peptide inducing T cell responses.

Meanwhile, for the patients with positive T cell responses in IFN-γ ELISPOT assay, HLA-A alleles were identified using polymerase chain reaction-sequencing-based typing (PCR-SBT) method, the gold-standard method recommended by the International HLA Work Group. Primers as described (25) were synthesized by Sangon Biotech Co., Ltd (Shanghai) and displayed in **Table S1**. The DNA from exon 1 to exon 3 of HLA-A was amplified in PCR using primer combination A1/A3 followed by sequencing using primer combination A2F/A2R for exon 2 and A3F/A3R for exon 3. The sequencing data were aligned with the sequences in the HLA database and analyzed using Lasergene software.

### 2.5 Peptide-PBMC Co-Culture Experiment Using Patient’s PBMCs

Briefly, PBMCs from the patients with HBV infection were prepared, then seeded in 96-well plates (4×10^5^ PBMCs/well) and incubated with a single validated epitope peptide (VEP) (20 μg/mL) which presented by the HLA-A allotypes of the indicated patient as *in silico* predicted, PHA (10 μg/mL) or no peptide in RPMI1640 culture medium with 10% FBS for 7 days at 37°C, 5% CO_2_ incubator. Recombinant human IL-2 (20 IU/mL) was added at day 3. On day 7, the corresponding peptide (40 μg/mL) or no peptide (negative control) was added again for another 16-hour co-culture. Then, BFA/Monensin mixture (eBioscience) was added to the cells and co-cultured for another 6 hrs. After that, cells were harvested, washed and blocked with anti-CD16/CD32 (eBioscience) for 20 min, then stained with FITC-labeled anti-human CD3 and APC-labeled anti-human CD8a monoclonal antibodies (mAbs, Biolegend) for 30 min at 4°C. After washing, cells were fixed and permeabilized following the protocol and were further stained with PE-labeled anti-human IFN-γ (BD Bioscience) for 30 min at 4°C followed by flow cytometry analysis. The frequencies of IFN-γ^+^ cells in CD3^+^/CD8^+^ populations were calculated.

### 2.6 Bioinformatics Analysis of Epitope Affinity With HLA-A Molecule

#### 2.6.1 Acquisition of HLA-A Structures for Simulation

The structure of HLA-A used for receptor was obtained in two ways. For HLA molecules with different binding conformations in world Protein Data Bank (PDB, http://www.wwpdb.org/), such as HLA-A1101, all the matching structures were collected using the mmseqs2 method in PDB (26). For HLA molecules without crystal structure in PDB, such as HLA-A3101, structures based on homology modeling were selected as receptors, using the Advanced Homology Modeling functionality in Biologics (Schrödinger 2020-4 release).

#### 2.6.2 Calculation of Free Energies of Binding

Since it is difficult to find the HLA-A structures presenting the HBV peptides, a reasonable docking conformation was obtained by the following two ways. As for structure collected from PDB, the peptide sequence in the complex with indicated HBV peptide was compared and the most similar complex based on the properties of main anchor residues was selected. Then, the original peptides in the peptide-HLA complex were mutated manually into the indicated HBV peptides. As for modelled structures, CodockPP server (27) (http://codockpp.schanglab.org.cn/index.php) was used to dock the peptides which derive from HLA modeling templates with them. The best docking poses according to Ligand RMSD are retained for subsequent scanning calculations (28). Some cases, such as HLA-A1101, due to the properties of the amino acids of its peptide, it cannot be mutated into HBV peptides. The peptide docking functionality in Biologics and molecular dynamics simulation functionality in Desmond (Schrödinger 2020-4 release) were used to get reasonable binding conformation. And the Generalized Bonn surface area based on molecular mechanics (MM-GBSA) in Prime (Schrödinger 2020-4 release) were used to obtain binding free energy.

Residue Scanning makes a specified list of mutations and then performs MM-GBSA calculations of the bound and unbound state for each system for both the wild type and the mutant. The predicted change in binding affinity is calculated using the equation and thermodynamic cycle as below:


$$\begin{array}{l} \Delta \Delta G_{bind}=\Delta G_{bind}^{WT}-\Delta G_{bind}^{WT}\\ \Delta \Delta G_{bind}=(E_{A\cdot B}^{MUT}-E_{A}^{MUT}-E_{B}^{MUT})-(E_{A\cdot B}^{WT}-E_{A}^{WT}-E_{B}^{WT})\\ \begin{array}{ccc} P_{A}+P_{B} & \overset{\Delta G_{bind}^{WT}}{\to } & P_{A}\cdot P_{B}\\ \Delta G_{Calc}^{Unbound}↓ & & ↓\Delta G_{Calc}^{Bound}\\ P_{A}^{'}+P_{B} & \underset{\Delta G_{bind}^{MUT}}{\to } & P_{A}^{'}\cdot P_{B} \end{array} \end{array}$$

Thermodynamic cycle for calculating the net ΔΔG free energy difference between binding the wild-type protein P and the mutant protein P’. E is the calculated energy of each protein or complex after refinement (29).

### 2.7 Generation of HMy2.CIR Cell Lines Expressing the Indicated HLA-A Molecule

HMy2.CIR cell line was purchased (Zhongqiao xinzhou Biotech, Shanghai) and maintained in complete IMDM medium with 10% FCS and 1% penicillin/streptomycin. Total mRNA was extracted from PBMCs of healthy donor with the indicated HLA-A alleles by RNAprep Pure Hi-Blood Kit (TIANGEN, Beijing, China), and the cDNA was generated by using HiScriptII1^st^ Strand cDNA Synthesis Kit (Vazyme, China) at RT. Each HLA-A allele was then amplified in PCR using the primer combination AF/AR, and the 5’ and 3’ ends of PCR product contained the same sequences as that of the linearized vector. Linearized pcDNATM3.1/myc-His (–)AMCS was amplified in PCR using the primer combination P-1F/P-1R. Finally, the routine construction of pcDNATM3.1 recombinant plasmids were obtained with ClonExpressII One Step Cloning Kit (Vazyme, China).

Primers were synthesized by Sangon Biotech (Shanghai) and displayed in **Table S1**. After electrotransfection, the cell lines stably expressing HLA-A molecule were screened by G418, and then stained with PE-anti-HLA-ABC (clone W6/32, eBioscience), FITC-anti-HLA-A24 (clone 17A10, MBL) or PE-anti-HLA-A2 (clone BB7.2, BD Bioscience). The resulting cells highly expressing the indicated HLA-A allotypes were positively sorted using a fluorescence activated cell sorter (FACS, BD FACSAriaIISORP), and followed by cell pure culture and gene sequencing analyses.

### 2.8 Peptide Competitive Binding Assay for HLA-A Molecules

A set of fluoresce-labeled reference peptides (positive-control peptide) were synthesized with a purity of >99% by Sangon Biotech and stored at 4°C, such as FLPSDK(FITC)FPSV for A0201, A0203 and A0206 (30), YVNVNK(FITC)GLK for A1101 and A3303 (31), EYLVSK(FITC)GVW for A2402 (32), ATFQFK(FITC)VER for A3101, A1102 and A0301 (33), KLPDDFK(FITC)GCV for A0207 (34), YLEPAK(FITC)AKY for A0101 (30), and ASRELK(FITC)VSY for A3001 (identified in house). Then the competitive peptide binding assay was performed as described with minor modifications (30, 35). Briefly, the HMy2.CIR cell lines expressing the indicated HLA-A molecule were washed with acid buffer (0.131M citric acid and 0.061M sodium phosphate Na_2_HPO_4_, PH3.3, 0.22 μm filtered) for 1 min, and immediately neutralized by IMDM medium containing 0.5% BSA. After washing, the cells were seeded into 96-well U culture plate (1×10^5^ cells/100 μL/well) with β_2_-m (1 μg/mL). Then 25 μL unlabeled competitor peptide (5 μM or 15 μM) and 25 μL corresponding FITC-labeled reference peptide (300 nM) were added into the well and co-incubated for 24 h at 4°C. In parallel, no peptide control well (cells alone) and positive control well (cells with FITC-labeled reference peptide) were also performed.

The plate was centrifuged at 600 rpm for 5 min at room temperature (RT). Cells were washed twice with 100 μL cold 0.5% BSA-PBS. Finally, cells were resuspended with 150 μL PBS and analyzed with flow cytometry. Sample % is the percentage of FITC^+^ cells in the experiment well, while the background % is the percentage of FITC^+^ cells in the no peptide control well, and the max % is the percentage of FITC^+^ cells in the positive control well. Competitive binding (%) = [1-(sample % - background %)/(max % – background %)] × 100%. IC50 is the concentration of unlabeled competitor peptide required to inhibit the binding of FITC-labeled reference peptide by 50%, which is calculated from the competitively binding inhibition (%) of the sample at 5 μM and 15 μM. Binding affinity of unlabeled peptide with the indicated HLA-A molecule is assessed by IC50. IC50<5μM (5μM inhibition >50%) means high binding affinity, 5μM<IC50<15μM (5μM inhibition<50%, but 15μM inhibition >50%) means intermediate binding affinity, IC50>15μM means low or no binding affinity (5μM inhibition 20-50% or 15μM inhibition 30-50% means low binding affinity; 5μM inhibition <20% or 15μM inhibition <30% means no binding affinity).

### 2.9 Clinical Detection of HBV-Specific T Cells for Patients With HBV Infection

The CD8^+^ T cell epitopes validated in house were integrated with the CD8^+^ T cell epitopes reported previously to establish an HBV-specific antigenic peptide library containing 105 epitopes. These epitopes are presented by 13 predominant HLA-A allotypes which have a total gene frequency of around 95% in Chinese and Northeast Asian populations and were arranged into seven peptide pools (15 epitopes/pool) to establish the pool-array IFN-γ ELISPOT assay for the enumeration of active/memory HBV-specific T cells in peripheral blood.

Fresh peripheral blood samples from in-patients with chronic hepatitis B were collected in the Division of Hepatitis at Nanjing Second Hospital and processed into PBMCs followed by IFN-γ ELISPOT assay according to manufacturer’s protocols (Dakewe Biotech). Notably, the PBMCs from each patient were co-cultured in 96-well plates with 7 peptide pools (2×10^5^ cells in each well with one peptide pool, 2 μg/peptide/well) for 20 hrs. In parallel, the positive control well with PHA (0.25 μg/well) and negative control well with PBMC alone were carried out. SFUs/2×10^5^ PBMCs = (Sum of actual spot numbers in the 7 experimental wells) - (Sum of background spot numbers in the 7 experimental wells). When the actual spot number in the indicated experimental well is less than that in the negative control well, the actual spot number is taken as the background spot number of the indicated experimental well. When the actual spot number in the indicated experimental well is equal to or greater than that in the negative control well, the spot number in the negative control well is taken as the background spot number in the indicated experimental well. The clinical baseline features and the real-time data from clinical laboratory were also collected for each patient.

### 2.10 Statistical Analysis

Statistical analyses were performed using GraphPad Prism 9 (GraphPad, La Jolla, CA, USA). Data were expressed as median (range) or mean (± standard error of mean, SEM). One-way analysis of variance (ANOVA) (for gaussian data) or Kruskal-Wallis test (for non-paprametric data) was performed when analyzing more than two groups. Then, a Mann-Whitney (non-parametric) test was used for the analysis of HBV DNA and Alanine aminotransferase (ALT) means, SFUs, HBsAg and HBeAg medians between two groups. The χ2 tests were used for the comparison of Anti-HBc IgM positive/negative categorical data across groups. Pearson (for gaussian data) correlation tests were performed to analyze correlation between peptide competitive binding experiment and bioinformatics analysis. For the analysis between stratified patient groups, multivariate linear regression analysis was performed. All statistical analyses were based on 2-tailed hypothesis tests with a significance level of *p*<0.05.

## 3 Results

### 3.1 187 CD8^+^ T Cell Epitopes Restricted by HLA-A Molecules Were *In Silico* Predicted From HBV Antigens and Selected as Candidate Epitopes

The potential epitopes derived from four HBV proteins (HBsAg, HBeAg, HBpol, HBx) and restricted, respectively, by 13 HLA-A allotypes were virtually predicted, by using epitope prediction tools and algorithms. The thirteen high-frequency HLA-A allotypes (HLA-A1101, A2402, A0201, A0207, A3303, A0206, A3001, A0203, A3101, A1102, A0101, A2601, A0301) cover over 95% Chinese and Northeast Asian populations with a gene frequency greater than 1% for each allele (http://www.allelefrequencies.net). For each HLA-A allotype and each protein, the top one to five 9- and 10-mer peptides with high affinity were chosen as best putative epitopes according to these criteria: the binding affinity exceeding the antigenic criteria of at least two algorithms; the ranking in top 1 to 5 as predicted by at least two algorithms. In addition, the diversities across virus genotypes and serotypes were also taken in account. Totally, 187 epitopes were selected as candidate epitopes with the number of 35, 28, 37 and 24 from HBsAg, HBeAg, HBpol, and HBx protein, respectively. Of the 187 predicted epitopes, 63 epitopes are common epitopes presented by several HLA-A allotypes as *in silico* predicted, so totally only 124 epitopes need to be synthesized as peptides for further investigation (**Table S2**).

### 3.2 Immunogenicity of 62 Candidate HBV Epitopes Was Validated by *Ex Vivo* ELISPOT Assay and *In Vitro* Co-Stimulation Using Patients’ PBMCs

**Figure 1:**
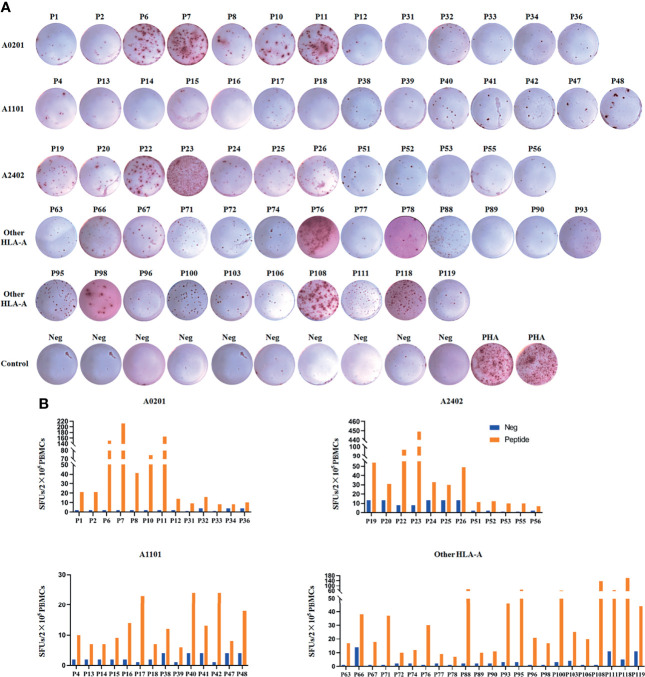
62 candidate HBV epitopes were validated as real-world T cell epitopes by IFN-γ ELISPOT assay using patients’ PBMCs. The PBMCs from 500 HBV infected patients were co-cultured 20 h with the peptide pools of 124 candidate epitopes. Of which, 106 PBMCs displayed positive T cell responses in the first-round IFN-γ ELISPOT assay. Then PBMCs were re-collected from 56 patients and co-cultured with single epitope peptide of the peptide pools positive in the first-round IFN-γ ELISPOT assay and followed by the second-round ELISPOT assay. **(A)** Representative SFU spot plots of 62 VEPs in the second-round IFN-γ ELISPOT assay. **(B)** SFUs histogram of each VEP and its negative control well. Neg, negative control well without peptide; PHA, positive control well with PHA.

Since HBV-specific CD8^+^ T cells can be detectable in only about 50% CHB patients’ PBMCs by *ex vivo* analyses as reported (36), here PBMCs were collected from a large cohort of HBV infected patients. During the 20-hour *ex vivo* co-incubation and ELISPOT assay, the candidate epitope peptide that can stimulate PBMCs to obviously produce IFN-γ was identified as validated epitope peptide (VEP), implying the presences of the peptide-specific memory or activated T cell clones in patient’s peripheral blood. Totally, 500 PBMCs samples were *ex vivo* tested with the peptide pools of 124 candidate epitopes, and 106 samples displayed positive T cell responses in the first-round ELISPOT assay. Of the 106 patients, 56 patients were re-enrolled and their PBMCs were re-collected followed by co-culture with each type of epitope peptides from the peptide pools which induced positive T cell responses in the first-round ELISPOT assay. After two rounds of ELISPOT assays, the immunogenicity of 62 candidate epitopes was finally validated (**Table 1**). The representative ELISPOT spot plots for each epitope were displayed in **Figure 1**. The clinical data were collected, and HLA-A genotyping were performed for the 56 patients (**Table S3**).

**Table 1:** 62 HBV epitopes and their HLA-A cross-restrictions validated by multiple approaches.

Epitope name	Protein	Epitope sequence	HBV genotype	Cons (%)	Predicted HLA restriction	ELISPOT positive	Activating CD8^+^ T cell	Reported HLA restriction	Bioinformatics analysis	Peptide competitive binding assay for HLA-A			
						Patient’s HLA	Patient’s HLA		Prime-affinity	High affinity	Inter affinity	Low affinity	No affinity
P2	HBsAg	FLLTRILTI	C/D	≥ 95	A0201, A0206 A0207	A0201/1136		A0201, A0205 A0206 (37)		A0201	A0207	A0206	A1101
P6	HBsAg	LLCLIFLLV	A/B/C/D	≥ 95	A0201, A0101	A0201/1136	A0201/0207A0201/3001	A02 (38)				A0207	A1101>A0101>A0201
P7	HBsAg	WLSLLVPFV	A/B/C/D	≥ 95	A0201, A0206 A0207, A0203	A0201/1136 A2402/2402 A1101/1101		A0201, A0202 A0206, A0203 A0207, A0205 (39)		A0203 >A0201>A0206		A0207	A1101>A2402
P8	HBsAg	GLSPTVWLSV	A/B/C/D	≥ 95	A0201	A0201/1136	A0201/3001	A0201 (40)				A0201	A1101
P13	HBsAg	SMYPSCCCTK	D	≥ 95	A1101, A1102 A0301	A0201/0206			A0201>A1101>A0301	A1101 >A0301>A1102			A0206
P14	HBsAg	ASPISSIFSR	C	80-95	A1101	A0206/1101			A1101			A1101	A0206
P19	HBsAg	LYSILSPFL	C/D	80-95	A2402	A2402/2402 A0201/1136 A0201/3201 A0201/0206			A2402	A2402			A0206>A0201>A1101
P20	HBsAg	LFILLLCLI	A/C/D	≥ 95	A2402	A2402/2402 A1101/1101			A2402				A1101>A2402
P38	HBsAg	SPISSIFSR	C	80-95	A0201, A1101	A0201/0201 A1101/3101			A0201>A1101				A1101>A3101>A0201
P39	HBsAg	SAISSISSK	B	< 80	A1101	A1101/0203			A1101>A0203	A1101			A0203
P40	HBsAg	QAGFFSLTK	B	< 80	A1101, A1102	A1101/3101	A1101/1101		A1101>A3101	A1101			A3101
P41	HBsAg	QAGFFLLTR	C/D	≥ 95	A1101, A1102	A1101/0201	A0201/2402		A1101>A1102				A1101>A1102>A0201
P51	HBsAg	VWLSVIWMMW	A/B/C/D	≥ 95	A2402	A0201/0207	A1101/1102		A2402>A0201>A0207		A2402	A0207	A0201
P71	HBsAg	MMWFWGPSL	B	80-95	A0207, A0301 A0101	A2402/2402 A2402/24109			A2402>A0207>A0301	A0207>A2402		A0301	A0101
P72	HBsAg	MMWYWGPSL	A/C/D	≥ 95	A0207, A1102 A0301, A0101	A0201/0201 A2402/0207			A0201>A0207>A2402	A0201>A2402>A0207		A0301	A0101>A1101>A1102
P77	HBsAg	CPGYRWMCLR	A/B/C/D	≥ 95	A3303	A0201/0201	A0201/3303		A3303				A3303>A0201
P78	HBsAg	FLWEWASVR	C	≥ 95	A3303	A0201/2402			A0201>A3303>A2402	A0201> A3303			A2402
P95	HBsAg	LLDYQGMLPV	A/B/C/D	≥ 95	A0203	A2402/2402 A2402/2402	A3303/3001	A02 (38)	A0203>A2402	A0203			A2402
P103	HBsAg	LQAGFFSLTK	B	< 80	A1102	A2402/0207			A0207>A2402>A1102	A1101		A0207	A1102>A2402
P108	HBsAg	MMWYWGPSLY	A/C/D	≥ 95	A0301, A0101 A1102	A2402/0207A2402/0207		A03 (37)	A2402>A0301>A0207			A0207>A1101>A0301	A0101>A2402>A1102
P1	HBeAg	FLPSDFFPSV	A/D	≥ 95	A0201, A0203 A0207, A0206	A0201/1136 A2402/3303 A1101/1101		A0201, A0203 A0206, A0207 (30)		A0201>A0206>A0203		A0207	A2402>A3303>A1101
P4	HBeAg	LVSFGVWIR	A/B/C/D	≥ 95	A1101, A1102	A1101/0206 A0201/0206		A33 (41)				A1101>A3303	A0206>A0201>A1102
P11	HBeAg	YLVSFGVWI	A/B/C/D	≥ 95	A0201, A0203	A0201/1136 A2402/1101 A2402/3303 A2402/0203	A0203/0203A1101/0301	A0201 (42)		A0203>A0201	A2402		A1101> A3303
P22	HBeAg	SYVNTNMGL	D	80-95	A2402	A2402/2402 A0206/1101		A0201 (32)		A2402			A1101>A0201> A0206
P23	HBeAg	EYLVSFGVW	A/B/C/D	≥ 95	A2402	A1101/1101 A0201/3201 A0207/2402		A2402, A2407 A2301 (43)		A2402			A0201>A1101
P31	HBeAg	FLPSDFFPSI	B/C	≥ 95	A0201, A0207 A0203	A0207/2402 A1101/2463	A1101/1101	A0201, A0207 (40)	A0207>A0201>A1101	A0203>A0201>A2402		A0207	A1101
P32	HBeAg	LLWFHISCL	A/B/C/D	≥ 95	A0201	A0201/2402	A2402/1101A0207/0207	A0201 (42)					A2402>A0201
P42	HBeAg	STLPETTVVR	A/B/C/D	/	A1101, A1102	A1101/0201 A1101/2463 A0207/3001	A1101/1102	A0201, A11 A6801 (37)	A1101>A0207	A1101		A0207	A1102>A0201>A3001
P52	HBeAg	WFHISCLTF	A/B/C/D	≥ 95	A2402	A2402/0207 A0201/0207			A2402>A0207>A0201	A2402		A0207	A0201
P53	HBeAg	SYVNVNMGL	A/B/C	80-95	A2402	A2402/0207	A0207/0206A0201/2402A0206/1101		A2402>A0207	A2402		A0207	
P67	HBeAg	FLPSDFFPS	A/B/C/D	≥ 95	A0206, A0201 A0207, A0203	A2402/2601 A2402/3303	A3303/3001	A02 (44)		A0206>A0201>A0203		A0207	A2402>A3303
P74	HBeAg	ILCWGELMNL	B/C	≥ 95	A0207	A2402/0207 A2402/3315			A0207>A2402		A0201	A0207	A2402>A3303
P88	HBeAg	ASRELVVSY	B/C	80-95	A3001	A0201/0201 A0206/0206 A0101/0206 A0101/0206 A0201/2601	A0201/0206		A0201>A3001>A0206	A3001	A0201		A0101>A0206
P118	HBeAg	ETVLEYLVSV	C	≥ 95	A2601	A1101/2402 A1101/2402 A0203/3001 A0201/1101 A0201/0207			A2601>A1101>A0201			A0207>A3001	A1101>A0203>A0201>A2402
P66	HBeAg	MQLFHLCLI	A/B/C/D	/	A0206	A0201/0201	A3303/3001	A0201 (16)		A0201	A0206		
P96	HBeAg	ILSTLPETTV	A/B/C/D	/	A0203	A0201/0201		A02 (45)	A0201>A0203	A0203	A0201		
P111	HBeAg	LLDTASALY	A/B/D	≥ 95	A0101, A1102	A2402/2402 A2402/0207	A1101/1101		A0101>A1102		A0101	A0207	A1101>A0201>A2402>A1102
P10	HBpol	FLLSLGIHL	A/B/C/D	≥ 95	A0201, A0206 A0207	A0201/1136 A0201/0201 A0206/0206 A2402/1101 A2402/3303 A1101/1101		A0201, A0206 A0202 (37)		A0201>A3303		A0207>A0206	A2402>A1101
P15	HBpol	KVTKYLPLDK	D	< 80	A1101	A0206/1101			A1101	A1101			A0206
P16	HBpol	PTYKAFLCK	C/D	< 80	A1101, A1102	A1101/0206	A0203/1101		A1101>A0206	A1101			A1102>A0206
P24	HBpol	KYTSFPWLL	A/B/C/D	≥ 95	A2402	A0201/0206		A2301, A2402 (46)		A2402			A0201>A0206
P25	HBpol	FYPKVTKYL	D	< 80	A2402	A1101/1101			A2402>A1101	A2402			A1101
P33	HBpol	KLIGTHNSV	B	< 80	A0201, A0203	A0201/0201 A1101/0203			A0201>A0203>A1101		A0201	A0203	A1101
P34	HBpol	KLIGTDNSV	A/C	80-95	A0201, A0203	A0201/2402	A1101/1101A3303/2601		A0201>A0203	A0201	A0203		A2402
P55	HBpol	LYSSTVPCF	B	< 80	A2402	A0201/3303	A0201/2402		A2402>A3303>A0201	A2402			A0201>A3303
P56	HBpol	LYSSTVPVF	C	≥ 95	A2402	A0201/0207			A2402>A0207	A2402		A0207	A0201
P76	HBpol	FLLAQFTSA	A/B/C/D	≥ 95	A0207, A0203	A0207/1101			A0203>A0207			A0207>A0203	A1101
P89	HBpol	RSRSGAKLI	B/C	80-95	A3001	A0201/0201 A0101/0206 A0206/0206	A0201/3001		A0201>A0206>A3001	A3001		A0201	A0206>A0101
P90	HBpol	RSRSGANIL	C/D	≥ 95	A3001	A0201/0201			A0201>A3001	A3001		A0201	
P98	HBpol	LLAQFTSAI	A/B/C/D	≥ 95	A0203	A0203/0301 A0203/0201		A0201 (42)	A0203	A0203		A0201	
P106	HBpol	TLWKAGILYK	A/B/C/D	≥ 95	A1102, A0301	A2402/2402 A2402/2402 A2402/2402		A03 (47)	A0301>A2402>A1102	A0301	A1101		A1102>A2402
P119	HBpol	HTAELLAACF	A/B/C/D	≥ 95	A2601	A2402/2402			A2402>A2601				A2402
P12	HBx	CLFKDWEEL	D	≥ 95	A0201	A0201/1136		A0201 (48)		A0201			A1101
P17	HBx	TVNAHQILPK	A/D	< 80	A1101, A1102 A0301	A1101/3001	A3303/2601		A1101>A3001	A1101		A3001	
P18	HBx	STTDLEAYFK	A/B/C/D	≥ 95	A1101	A0201/0206 A0206/1101			A0201>A1101>A0206			A1101	A0206>A0201
P26	HBx	VCAPAPCNF	D	80-95	A2402	A0206/1101 A2402/2402	A1101/3303		A2402				A1101> A2402>A0206
P36	HBx	VLGGCRHKL	A/B/C/D	≥ 95	A0201	A1101/3101		A0201 (49)				A0201	A1101>A3101
P47	HBx	TVNAHGNLPK	B	< 80	A1101, A1102 A0301	A1101/0201			A0201>A1101>A0301	A1101>A0301		A1102	A0201
P48	HBx	TVNAHQVLPK	C	< 80	A1101, A1102 A0301	A1101/0201	A1101/1102		A1101	A1101>A0301		A1102	A0201
P63	HBx	KVFVLGGCR	A/B/C/D	< 80	A3101, A0301	A2402/2402	A1101/1101A3303/2601		A2402>A3101		A3101		A2402
P93	HBx	RLKVFVLGG	A/B/C/D	< 80	A3001	A2402/2402			A2402>A3001				A3001>A2402
P100	HBx	HLSLRGLPV	A/B/C/D	≥ 95	A0203	A2402/2402 A2402/2402 A2402/2402	A1101/1101	A0201 (49)		A0203			A2402

ELISPOT positive, the epitope peptide induced positive T cell response in the ex vivo IFN-γ ELISPOT assay with the indicated patient’s PBMCs. Activating CD8^+^ T cell, the epitope peptide induced positive CD8^+^ T cell response in the in vitro cocultures with the indicated patient’ PBMCs. Cons (%), conservation (%).

**Figure 2:**
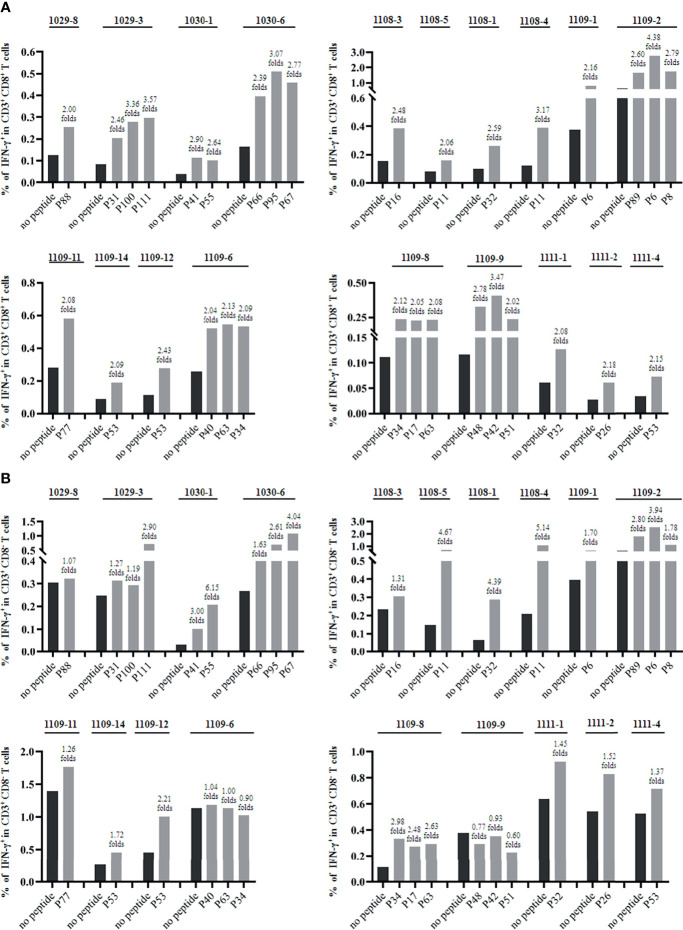
The validated HBV epitope peptides induced CD8^+^ T cell responses in the *in vitro* cocultures with patients’ PBMCs. The PBMCs were stimulated with the indicated epitope peptides for 7 days *in vitro* and followed by IFN-γ intracellular staining and flow cytometry. Totally, 25 epitope peptides were tested using the PBMCs from 19 patients with matching HLA-A alleles. **(A)** Each epitope peptide induced positive CD8^+^ T cell responses in the PBMCs from one, two or three patients. The frequency of IFN-γ^+^ T cells in CD3^+^/CD8^+^ T cell population for each VEPs and each responded patient was presented as histograms. **(B)** 13 of the 25 CD8^+^ T cell epitope peptides simultaneously induced CD4^+^ T cell responses. The frequency of IFN-γ^+^ T cells in CD3^+^/CD8^-^ T cell population for each VEPs and each responded patient was presented as histograms.

Furthermore, the epitopes validated by IFN-γ ELISPOT assay were assessed for their *in vitro* capacity to induce CD8^+^ T cell activation by co-cultures with patients’ PBMCs for 7 days and followed by IFN-γ intracellular staining. The frequencies of IFN-γ^+^ cells in CD3^+^/CD8^+^ or CD3^+^/CD8^-^ populations were analyzed by flow cytometry. When the frequency of IFN-γ^+^ cells in the co-culture well was two-fold greater than that of negative control well (PBMCs alone), the epitope peptide in the coculture was defined as the immunogenic epitope that can induce CD8^+^ or CD4^+^ T cell responses as described (50). Totally, 25 epitope peptides were tested using the PBMCs from 19 patients with matching HLA-A alleles. Each epitope peptide was confirmed to induce positive CD8^+^ T cell responses in the PBMCs from one, two or three patients. But interestingly, 13 of the 25 CD8^+^ T cell epitope peptides simultaneously induced CD4^+^ T cell responses 5with a two- to six-fold increase in IFN-γ^+^/CD3^+^/CD8^-^ T cell frequency in the *in vitro* peptide-PBMCs cocultures (**Figures 2**, **S2**).

### 3.3 Conservative Properties of 62 Validated HBV Epitopes Were Analyzed

The entire amino acid sequences of HBsAg, HBeAg, HBx and HBpol proteins from HBV C, B, A and D genotypes were screened for the CD8^+^ T cell epitopes restricted by the thirteen prevalent HLA-A allotypes in China. These sequences were obtained from UniProt database and derived from the HBV strains reported in China or Northeast Asia. In the *in silico* prediction, the overall sequences of target protein from each HBV genotype were screened. The distinct epitopes between different genotypes and serotypes were also selected as candidate epitope peptides for further validation (**Table S2**). The fourth column in **Table 1** displayed the validated common epitopes and distinct epitopes from different genotypes.

Furthermore, the consensus sequences of HBsAg, HBeAg, HBx and HBpol proteins and the conservative properties of 62 validated epitopes in this study were analyzed. Briefly, a huge number of sequences of HBsAg (n = 1410, 2802, 3124, 1493), HBeAg (n = 2168, 2542, 2669, 1375), HBpol (n = 1160, 2467, 3131, 1276) and HBx (n = 996, 2561, 3682, 1752) for HBV genotype A, B, C, and D were collected from the publicly available HBVdb database (http://hbvdb.ibcp.fr/) (51). Multiple sequence alignments were performed using the ClustalW method in the Molecular Evolutionary Genetics Analysis software (MEGA7, version 1.0.0.0) (52), and positions where a gap was neglected. Then, GeneDoc software (version 2.7.0) was used to analyze the alignment results to obtain the conservative region of these sequences. The conservative properties of each amino acid in each protein were judged by the threshold of 100%, 95%, 80%, and the amino acids were highlighted in different colors (100%: red, ≥95%: yellow, ≥80%: black in gray background, and <80%: black in white background) (**Figure S3**). Finally, 62 epitopes validated in this study were classified into three categories according to its conservation (≥95%, 80-95%, <80%) and displayed in the fifth column of **Table 1**. Conservation ≥95%: each amino acid in the epitope with the conservative properties of 95-100%; Conservation 80-95%: at least one amino acid in the epitope with the conservative properties of ≥80% and <95%; Conservation <80%: at least one amino acid in the epitope with the conservative properties of <80%. Of 62 validated epitopes, 10 epitopes in HBpol and 3 epitopes in HBsAg have the conservative properties of <80%.

### 3.4 Binding Affinity and Cross-Binding of 45 Validated HBV Epitopes With HLA-A Allotypes Were Analyzed by Bioinformatics Analysis

To further identify HLA-A restrictions of the validated peptides, the binding affinity between VEPs and corresponding HLA-A allotypes were analyzed by using bioinformatics methods, such as residue scanning, molecular docking and dynamic simulation. The three-dimensional structures of HLA-A allotypes were selected or constructed from PDB, which would be used as protein-protein interaction analysis. Then the binding motifs of all peptide-HLA complexes were analyzed. The peptide sequence in the complex with the indicated HBV peptide was compared and the most similar complex based on the properties of main anchor residues was selected. For HLA-A molecules that do not have peptide binding conformation, peptide docking, and dynamics simulation were used to achieve a reasonable binding conformation. Then physics-based scoring was used to calculate free energies of binding for peptide-HLA complex, which could characterize the strength of the bonding. In general, the greater the negative value is, the stronger the bond is. Δ Affinity values of less than 3 Kal/mol were considered to have a better affinity than the Bond Peptide in PDB of HLA. If the Δ Affinity score was greater than 3 Kal/mol, it would be considered that the HBV peptide might not bind to the corresponding HLA-A molecule (29). While Prime Affinity and MM-GBSA score represented the affinity score of HBV peptide to HLA-A molecule, it was generally believed that the smaller the score, the higher the Affinity, which could be used for ranking (29). The representative schematic diagrams of the interaction between HBV peptide and HLA-A molecule were displayed in **Figure S4**. Here, 45 HBV epitope peptides validated in house were analyzed for their cross-binding affinity with corresponding HLA-A allotypes. Each peptide was tested with several HLA-A allotypes. The calculation results were displayed in **Table S4** and ranked in **Table 1**.

### 3.5 Binding Affinity and Cross-Binding of 62 Validated HBV Epitope Peptides With HLA-A Allotypes Were Analyzed by Peptide Competitive Binding Assay

HMy2.CIR is a human B lymphocyte strain with HLA class I antigen deficiency, which does not express HLA-A and B molecules and only expresses trace HLA-Cw4. To assess the affinity of validated peptides with the corresponding HLA-A molecules, the transfected HMy2.CIR cell lines expressing the indicated HLA-A allotypes (HLA-A2402, A0201, A0203, A0206, A1101, A3303, A0101, A3001, A0207, A3101, A1102, or A0301) were generated firstly, sorted by flow cytometry, and identified by gene sequencing. The purity of these transfected HMy2.CIR cell lines was 80% to 94% after sorting (**Figure S5**).

Subsequently, the unlabeled epitope peptides of HBV competed with fluorescently labeled reference peptides in the binding with HLA-A molecules onto transfected cell lines for 24 hours. Here, twelve prevalent HLA-A allotypes were tested with totally 62 HBV epitope peptides validated in house. As shown in the line diagrams of flow cytometry, most competitor peptides could result in a left shift of the cell fluorescent peak from reference peptide (**Figure S6**), implying the efficient binding of epitope peptides with associated HLA-A molecules. **Table 2** exhibited the binding affinity of each peptide with associated HLA-A allotypes. Notably, most epitope peptides displayed cross-reactive behavior with several HLA-A allotypes. Especially, the P1, P7, P13, P31, P72 or P67 bound to three HLA-A allotypes with high affinity while P10, P11, P47, P48, P71 or P78 bound to two HLA-A allotypes with high affinity. Totally, 58 high-affinity, 12 intermediate-affinity and 39 low-affinity epitopes were identified in the *in vitro* experiments. Most peptides with high or intermediate binding affinity displayed conservative amino acids at P2 position and COOH terminus (**Table 2**).

**Table 2:** Binding affinity of 62 HBV T cell epitopes with twelve prevalent HLA-A allotypes as detected by peptide competitive binding experiments using transfected Hmy2. CIR cell lines.

HLA-A	Peptides	Affinity	5μM Inhibition (%)	15μM Inhibition (%)	Sequence
A0201	P1	high	94.60%	98.20%	F**L**PSDFFPS**V**
	P67	high	91.53%	97.83%	F**L**PSDFFP**S**
	P72	high	88.70%	93.40%	M**M**WYWGPS**L**
	P7	high	83.00%	96.40%	W**L**SLLVPF**V**
	P10	high	81.70%	71.60%	F**L**LSLGIH**L**
	P11	high	81.20%	96.20%	Y**L**VSFGVW**I**
	P2	high	80.00%	87.80%	F**L**LTRILT**I**
	P78	high	79.72%	97.30%	F**L**WEWASV**R**
	P31	high	79.00%	90.40%	F**L**PSDFFPS**I**
	P66	high	70.10%	83.50%	M**Q**LFHLCL**I**
	P12	high	64.30%	80.40%	C**L**FKDWEE**L**
	P34	high	50.00%	52.30%	K**L**IGTDNS**V**
	P88	inter	47.90%	58.60%	A**S**RELVVS**Y**
	P33	inter	37.90%	50.00%	K**L**IGTHNS**V**
	P74	inter	33.15%	74.09%	I**L**CWGELMN**L**
	P96	inter	20.50%	51.00%	I**L**STLPETT**V**
	P89	low	25.80%	27.10%	RSRSGAKLI
	P36	low	22.30%	37.30%	VLGGCRHKL
	P90	low	19.60%	28.70%	RSRSGANIL
	P98	low	15.26%	32.81%	LLAQFTSAI
	P8	low	6.70%	30.50%	GLSPTVWLSV
	P24	no	13.00%	12.30%	KYTSFPWLL
	P22	no	11.30%	11.00%	SYVNTNMGL
	P23	no	10.76%	8.18%	EYLVSFGVW
	P111	no	10.60%	14.60%	LLDTASALY
	P48	no	9.50%	15.00%	TVNAHQVLPK
	P18	no	9.40%	10.00%	STTDLEAYFK
	P55	no	8.20%	11.60%	LYSSTVPCF
	P118	no	7.95%	11.78%	ETVLEYLVSV
	P4	no	7.90%	11.90%	LVSFGVWIR
	P52	no	7.39%	7.05%	WFHISCLTF
	P47	no	7.20%	12.00%	TVNAHGNLPK
	P42	no	6.20%	14.20%	STLPETTVVR
	P56	no	5.48%	5.03%	LYSSTVPVF
	P41	no	5.00%	11.40%	QAGFFLLTR
	P19	no	3.60%	13.10%	LYSILSPFL
	P51	no	2.00%	4.50%	VWLSVIWMMW
	P6	no	0.00%	14.80%	LLCLIFLLV
	P32	no	0.00%	0.00%	LLWFHISCL
	P38	no	0.00%	0.00%	SPISSIFSR
	P77	no	0.00%	0.00%	CPGYRWMCLR
A1101	P103	high	89.10%	89.40%	L**Q**AGFFSLT**K**
	P40	high	87.66%	92.84%	Q**A**GFFSLT**K**
	P48	high	86.00%	90.95%	T**V**NAHQVLP**K**
	P39	high	85.95%	92.42%	S**A**ISSISS**K**
	P13	high	79.48%	83.91%	S**M**YPSCCCT**K**
	P47	high	78.34%	90.30%	T**V**NAHGNLP**K**
	P17	high	65.39%	84.36%	T**V**NAHQILP**K**
	P16	high	61.41%	79.25%	P**T**YKAFLC**K**
	P15	high	60.16%	78.80%	K**V**TKYLPLD**K**
	P42	high	53.80%	76.98%	S**T**LPETTVV**R**
	P106	inter	20.00%	91.30%	T**L**WKAGILY**K**
	P14	low	36.18%	45.27%	ASPLSSIFSR
	P108	low	31.80%	32.00%	MMWYWGPSLY
	P4	low	30.05%	35.95%	LVSFGVWIR
	P18	low	23.68%	41.30%	STTDLEAYFK
	P11	no	17.10%	20.00%	YLVSFGVWI
	P26	no	15.30%	22.40%	VCAPAPCNF
	P12	no	14.90%	22.60%	CLFKDWEEL
	P72	no	14.30%	14.70%	MMWYWGPSL
	P8	no	14.10%	11.50%	GLSPTVWLSV
	P36	no	14.00%	20.60%	VLGGCRHKL
	P6	no	13.50%	10.00%	LLCLIFLLV
	P118	no	13.40%	14.60%	ETVLEYLVSV
	P38	no	13.23%	14.59%	SPISSIFSR
	P22	no	12.00%	16.20%	SYVNTNMGL
	P41	no	11.98%	12.89%	QAGFFLLTR
	P111	no	11.00%	14.80%	LLDTASALY
	P23	no	8.91%	1.64%	EYLVSFGVW
	P31	no	8.50%	9.50%	FLPSDFFPSI
	P33	no	8.00%	14.80%	KLIGTHNSV
	P10	no	7.20%	8.00%	FLLSLGIHL
	P7	no	5.50%	3.11%	WLSLLVPFV
	P20	no	5.05%	4.48%	IFILLLCLI
	P2	no	4.50%	8.50%	FLLTRILTI
	P25	no	4.14%	9.48%	FYPKVTKYL
	P1	no	1.41%	5.95%	FLPSDFFPSV
	P19	no	0.90%	16.20%	LYSILSPFL
	P76	no	0.00%	0.00%	FLLAQFTSA
A3303	P78	high	72.10%	89.60%	F**L**WEWASV**R**
	P10	high	60.14%	66.07%	F**L**LSLGIH**L**
	P4	low	27.37%	33.02%	LVSFGVWIR
	P11	no	10.25%	4.63%	YLVSFGVWI
	P1	no	7.10%	10.02%	FLPSDFFPSV
	P77	no	6.50%	10.70%	CPGYRWMCLR
	P55	no	0.00%	0.00%	LYSSTVPCF
	P67	no	0.00%	0.00%	FLPSDFFPS
	P74	no	0.00%	0.00%	ILCWGELMNL
A0203	P96	high	100.00%	99.40%	I**L**STLPETT**V**
	P95	high	97.70%	95.95%	L**L**DYQGMLP**V**
	P7	high	96.30%	98.70%	W**L**SLLVPF**V**
	P11	high	89.40%	97.40%	Y**L**VSFGVW**I**
	P100	high	88.60%	98.90%	H**L**SLRGLPV
	P1	high	87.34%	92.11%	F**L**PSDFFPS**V**
	P31	high	86.26%	90.92%	F**L**PSDFFPS**I**
	P67	high	86.26%	91.52%	F**L**PSDFFP**S**
	P98	high	52.37%	82.68%	L**L**AQFTSA**I**
	P34	inter	41.52%	69.32%	K**L**IGTDNS**V**
	P33	low	20.63%	41.87%	KLIGTHNSV
	P76	low	20.39%	36.86%	FLLAQFTSA
	P118	no	11.92%	17.05%	ETVLEYLVSV
	P39	no	2.61%	3.81%	SAISSISSK
A0206	P67	high	95.70%	100.00%	F**L**PSDFFP**S**
	P1	high	94.39%	100.91%	F**L**PSDFFPS**V**
	P7	high	82.90%	96.20%	W**L**SLLVPF**V**
	P66	inter	27.30%	51.60%	M**Q**LFHLCL**I**
	P2	low	22.43%	39.90%	FLLTRILTI
	P10	low	21.50%	43.20%	FLLSLGIHL
	P89	no	15.60%	12.10%	RSRSGAKLI
	P4	no	15.12%	14.21%	LVSFGVWIR
	P19	no	14.86%	10.56%	LYSILSPFL
	P13	no	12.13%	10.17%	SMYPSCCCTK
	P18	no	10.82%	13.43%	STTDLEAYFK
	P15	no	10.56%	9.91%	KVTKYLPLDK
	P24	no	9.39%	9.13%	KYTSFPWLL
	P16	no	9.00%	8.08%	PTYKAFLCK
	P26	no	8.08%	7.69%	VCAPAPCNF
	P22	no	7.95%	11.21%	SYVNTNMGL
	P14	no	7.17%	8.87%	ASPISSIFSR
	P88	no	0.00%	0.60%	ASRELVVSY
A2402	P24	high	89.50%	95.30%	K**Y**TSFPWL**L**
	P56	high	85.32%	87.32%	L**Y**SSTVPV**F**
	P25	high	82.50%	83.60%	F**Y**PKVTKY**L**
	P19	high	82.00%	85.00%	L**Y**SILSPF**L**
	P55	high	78.21%	90.82%	L**Y**SSTVPC**F**
	P23	high	76.15%	85.91%	E**Y**LVSFGV**W**
	P22	high	72.20%	85.00%	S**Y**VNTNMG**L**
	P31	high	70.60%	86.10%	F**L**PSDFFPS**I**
	P53	high	68.50%	74.68%	S**Y**VNVNMG**L**
	P52	high	58.21%	75.85%	W**F**HISCLT**F**
	P72	high	55.70%	71.40%	M**M**WYWGPS**L**
	P71	high	50.32%	62.91%	M**M**WFWGPS**L**
	P11	inter	41.00%	52.50%	Y**L**VSFGVW**I**
	P51	inter	39.97%	52.50%	V**W**LSVIWMM**W**
	P10	no	19.00%	17.00%	FLLSLGIHL
	P34	no	11.40%	12.00%	KLIGTDNSV
	P100	no	10.26%	14.68%	HLSLRGLPV
	P111	no	10.26%	19.09%	LLDTASALY
	P1	no	10.00%	25.00%	FLPSDFFPSV
	P63	no	9.68%	4.38%	KVFVLGGCR
	P26	no	9.68%	29.09%	VCAPAPCNF
	P108	no	4.50%	8.00%	MMWYWGPSLY
	P119	no	3.10%	15.85%	HTAELLAACF
	P93	no	2.62%	18.79%	RLKVFVLGG
	P74	no	2.20%	11.10%	ILCWGELMNL
	P106	no	2.10%	16.15%	TLWKAGILYK
	P67	no	2.00%	4.30%	FLPSDFFPS
	P95	no	1.74%	19.38%	LLDYQGMLPV
	P103	no	1.60%	4.50%	LQAGFFSLTK
	P78	no	1.00%	9.10%	FLWEWASVR
	P20	no	0.90%	1.30%	IFILLLCLI
	P32	no	0.70%	0.70%	LLWFHISCL
	P118	no	0.50%	3.90%	ETVLEYLVSV
	P7	no	0.00%	0.00%	WLSLLVPFV
A3001	P88	high	69.00%	74.75%	A**S**RELVVS**Y**
	P90	high	68.00%	70.00%	R**S**RSGANI**L**
	P89	high	62.25%	71.75%	R**S**RSGAKL**I**
	P17	low	42.50%	46.50%	TVNAHQILPK
	P118	low	24.00%	26.50%	ETVLEYLVSV
	P93	no	15.75%	24.00%	RLKVFVLGG
	P42	no	4.50%	4.25%	STLPETTVVR
A0101	P111	inter	31.22%	62.33%	L**L**DTASAL**Y**
	P108	no	11.22%	13.78%	MMWYWGPSLY
	P89	no	10.11%	9.00%	RSRSGAKLI
	P6	no	9.44%	10.11%	LLCLIFLLV
	P71	no	9.00%	7.89%	MMWFWGPSL
	P72	no	9.00%	9.00%	MMWYWGPSL
	P88	no	9.00%	9.00%	ASRELVVSY
A0207	P71	high	52.00%	59.00%	M**M**WFWGPS**L**
	P72	high	52.00%	54.00%	M**M**WYWGPS**L**
	P2	inter	36.00%	52.00%	F**L**LTRILT**I**
	P31	low	47.00%	45.00%	FLPSDFFPSI
	P74	low	45.00%	43.00%	ILCWGELMNL
	P51	low	43.00%	43.00%	VWLSVIWMMW
	P67	low	42.00%	48.00%	FLPSDFFPS
	P76	low	41.00%	41.00%	FLLAQFTSA
	P118	low	40.00%	39.00%	ETVLEYLVSV
	P1	low	39.00%	45.00%	FLPSDFFPSV
	P103	low	39.00%	44.00%	LQAGFFSLTK
	P7	low	38.00%	25.00%	WLSLLVPFV
	P108	low	38.00%	40.00%	MMWYWGPSLY
	P111	low	37.00%	39.00%	LLDTASALY
	P52	low	36.00%	38.00%	WFHISCLTF
	P53	low	36.00%	31.00%	SYVNVNMGL
	P42	low	33.00%	45.00%	STLPETTVVR
	P56	low	32.00%	36.00%	LYSSTVPVF
	P6	low	31.00%	22.00%	LLCLIFLLV
	P10	low	27.00%	31.00%	FLLSLGIHL
A3101	P63	inter	35.43%	55.43%	K**V**FVLGGC**R**
	P40	no	4.93%	5.48%	QAGFFSLTK
	P36	no	4.82%	5.70%	VLGGCRHKL
	P38	no	4.04%	4.49%	SPISSIFSR
A1102	P13	high	54.67%	73.07%	S**M**YPSCCCT**K**
	P47	low	24.71%	44.19%	TVNAHGNLPK
	P48	low	25.52%	42.71%	TVNAHQVLPK
	P103	no	18.94%	25.12%	LQAGFFSLTK
	P16	no	13.7%	27.27%	PTYKAFLCK
	P42	no	12.36%	25.52%	STLPETTVVR
	P106	no	8.19%	8.19%	TLWKAGILYK
	P72	no	7.12%	9.54%	MMWYWGPSL
	P41	no	6.72%	9.40%	QAGFFLLTR
	P111	no	0.27%	0.00%	LLDTASALY
	P4	no	0.00%	0.00%	LVSFGVWIR
	P108	no	0.00%	0.00%	MMWYWGPSLY
A0301	P13	high	79.85%	80.7%	S**M**YPSCCCT**K**
	P106	high	78.14%	82.84%	T**L**WKAGILY**K**
	P48	high	67.89%	73.87%	T**V**NAHQVLP**K**
	P47	high	65.76%	78.14%	T**V**NAHGNLP**K**
	P72	low	26.9%	35.87%	MMWYWGPSL
	P108	low	26.05%	29.46%	MMWYWGPSLY
	P71	low	22.2%	38.00%	MMWFWGPSL

Binding affinity of epitope peptide with indicated HLA-A allotype was assessed by IC_50,_ which is the concentration of unlabeled competitor peptide required to inhibit the binding of fluoresce-labeled reference peptide by 50%. IC_50_<5μM (5μM inhibition >50%) means high binding affinity, 5μM<IC50<15μM (5μM inhibition<50%, but 15μM inhibition >50%) means intermediate binding affinity, IC_50_>15μM means low or no binding affinity. 5μM inhibition 20-50% or 15μM inhibition 30-50% means low binding affinity; 5μM inhibition <20% or 15μM inhibition <30% means no binding affinity.

Most peptides with high or intermediate binding affinity displayed conservative amino acids at P2 position and COOH terminus (bold letter).

**Figure 3:**
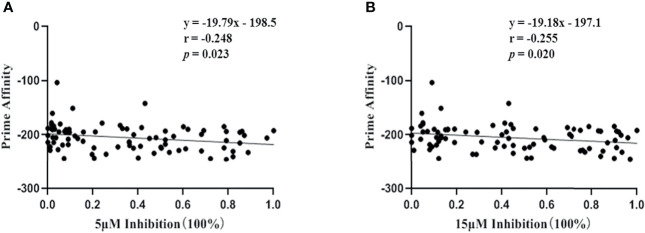
Correlation coefficient between bioinformatics analysis and peptide competitive binding assay for HLA-A molecules. Vertical axis represented the prime affinity of 45 validated epitope with corresponding HLA-A allotypes calculated by bioinformatics analysis, while horizontal axis represented the binding affinity of identical validated epitopes with corresponding HLA-A allotypes detected by peptide competitive binding assay for HLA-A molecules using transfected HMy2.CIR cell lines. The percentages of inhibition efficacy in peptide competitive binding assay were transformed into 0 to 1.0 for the Pearson correlation analysis (for gaussian data). **(A)** Correlation of bioinformatics analysis with the peptide competitive binding assay under 5μM of VEP. **(B)** Correlation of bioinformatics analysis with the peptide competitive binding assay under 15μM of VEP. *p*<0.05 means that the correlation analysis had statistical significance.

In addition, although results from peptide competitive binding experiments were partially inconsistent with the data from bioinformatics analysis (**Table 1**), the correlation coefficient between the two methods was confirmed, r=-0.248 at 5 μM inhibition or -0.255 at 15 μM inhibition, *p <*0.05 (**Figure 3**).

### 3.6 HBV Specific T Cells Were Detected for Patients With HBV Infection by Using 105 CD8^+^ T Cell Epitope Peptides and IFN-γ ELISPOT Assay

In order to monitor the HBV-specific cellular immune function for the broad patients carrying distinct HLA-A alleles, 62 epitopes validated here were integrated with other 43 CD8^+^ T cell epitopes which were mostly presented by the relatively low-frequency HLA-A allotypes (A0101, A1102, A2601 and A0301) and reported by other researchers or *in silico* predicted but confirmed by peptide competitive binding experiments using transfected HMy2.CIR cell lines in this study. Of these epitopes, each one can be cross-bound by several ones of the 13 most predominant HLA-A allotypes in Chinese and Northeast Asian populations. The validated epitope peptides restricted by each HLA-A allotype were exhibited in **Table 3**. Meanwhile, we cannot exclude the possibility that these antigenic peptides can also be presented by other HLA-A allotypes beyond the 13 prevalent ones here.

**Table 3:** Antigen distribution and HLA-A restrictions of 105 HBV T cell epitopes used in the clinical detection of HBV-specific T cells.

HLA-A allotype	Allele frequency	HBV CD8^+^ T cell epitope peptides				Total
		HBsAg	HBeAg (covering HBcAg)	HBpol	HBx	
A1101	22.4%	P101, P103, P107, P108, P13, P14, P38, P39, P40, P41	P109, P118, P1, P23, P4, P31, P42, P43	P105, P106, P110, P10, P15, P16, P46, P25, P33	P17, P47, P48, P18, P83, P84	33
A2402	15.3%	P19, P51, P20, P78, P108, P103, P71, P72, P95	P23, P22, P53, P52, P111, P31, P11, P67, P74	P24, P56, P25, P55, P106, P119	P63, P93, P100, P26	28
A0201	13.9%	P72, P78, P13, P7, P2, P8, P38, P77, P19	P1, P67, P11, P31, P32, P66, P88, P96, P118, P52, P74	P68, P10, P33, P55, P34, P99, P98, P89, P90	P18, P47, P12, P36	33
A0207	9.5%	P71, P72, P2, P51, P108, P73, P103	P31, P74, P67, P118, P1, P111, P52, P53,P42	P56, P10, P76	P35	20
A3303	8.1%	P78, P77, P59, P58, P79, P122	P43, P4, P80	P10, P55, P81, P82	P84, P83	15
A0206	6.1%	P7, P2	P1, P67, P66, P88	P68, P10, P99, P16, P89	P69, P70	13
A3001	5.2%	P85, P86	P87, P88, P118	P91, P90, P89	P93, P94, P92, P17	12
A0203	3.9%	P95, P7, P39	P96, P11, P1, P31, P67, P97	P99, P98, P34, P68, P33, P76	P100, P35	17
A3101	3.4%	P58, P40, P59	P43, P60	P61, P62	P63, P84, P64	10
A1102	2.3%	P40, P41, P72, P108, P13, P101, P102, P103	P111, P4, P42, P104, P109	P106, P16, P105	P17, P47, P48, P83, P121	21
A0101	2.2%	P72, P71, P108, P6	P111	P112, P113	P114	8
A2601	1.8%	P115, P116	P118, P117	P119, P113, P120	P121	8
A0301	1.4%	P71, P72, P101, P108, P13, P107	P43, P109, P104	P110, P106	P17, P48, P63, P47	15

Then, IFN-γ ELISPOT assay using the 105 validated peptides was performed to enumerate the memory or active HBV-specific T cells (mainly CD8^+^ T cells) in the peripheral blood of 116 patients with HBV infection. The clinical baseline features of enrolled patient cohort were displayed in **Table 4**. In order to assess the correlation of HBV-specific T cell response with clinical parameters, patients were grouped into three subsets according to IFN-γ^+^ SFU levels: 25% of the cohort with low SFU level (0-19), 50% of the cohort with intermediate SFU level (20-73), and 25% of the cohort with high SFU level (74-502). As shown in **Table 5**, serum HBeAg levels were detected lower in the inter-level SFUs group than in the high-level SFUs group (Median:0.12 *vs* 2.87, p=0.023). No significant difference on serum viral load, ALT, HBsAg, or anti-HBc IgM parameters was found across the SFUs groups. Moreover, multivariate linear regression analysis was performed for these items. The SFUs (assigned: continuous variable) was used as the dependent variable, while HBV DNA, ALT, HBsAg, HBeAg and Anti-HBc-IgM (all as classification variables) were used as independent variables. The results showed that the regression equation was tested as F = 3.112, p = 0.004. indicating that the regression model was qualified. The collinearity was evaluated by variance inflation factor. The P values of DNA, ALT, HBsAg, HBeAg and Anti-HBc-IgM were 0.300, 0.898, 0.000, 0.834 and 0.756, respectively. HBsAg levels significantly correlated with SFUs levels. In addition, age, different diseases (R, CHB, LC and HCC) and different stages of CHB (IT, IA and IC) were also used as independent variables in different regression models, no correlation with SFUs levels was found.

**Table 4:** Baseline features of 116 HBV infected patients enrolled in this study [median (min-max)].

Clinical Features	R	CHB	LC	HCC
Number	29	53	20	14
Age (years)	60 (25-89)	41.0 (17-81)	49 (32-66)	59 (34-71)
Gender (male/female)	17/12	30/23	19/5	10/4
ALT (IU/L)	17.9 (4.7-110.8)	57.15 (6.7-2260)	22.8 (7.9-96.9)	36.65 (15.2-701.6)
HBV DNA (log_10_ IU/mL)	No test	3.67 (2.6-8.89)	2.6 (2.6-6.78)	2.6 (2.6-6.62)
HBsAg (IU/mL)	< 0.05	1844 (0.61-52000)	372.8 (0.23-1991)	232.5 (2.28-1886)
HBsAb (mIU/mL)	28.3 (2.0-1000)	2.0 (0.06-119.1)	2.0 (0.06-110.4)	2.6 (2.0-467.2)
HBeAg (COI)	0.11 (0.09-0.14)	14.08 (0.09-1909)	0.39 (0.09-74.48)	0.205 (0.09-71.07)
HBeAb (COI)	0.83 (0.00-1.45)	1.10 (0.00-65.75)	0.44 (0.0-1.49)	0.715 (0.0-1.35)
HBcAb (COI)	0.01 (0.01-0.82)	0.01 (0.01-10.74)	0.01 (0.01-0.12)	0.01 (0.01-0.01)

R, acute resolved patients; CHB, chronic hepatitis B virus; LC, HBV-related liver cirrhosis; HCC, HBV-related hepatocellular carcinoma.

COI, cut off index, COI = sample value/cut off value. COI >1.0 means positive result, COI<1.0 means negative result.

**Table 5:** Stratification analysis of HBV-specific T cell responses for 116 HBV infected patients.

	ELISPOT SFUs	HBV DNA (log_10_ IU/ml) Mean ± SEM	n	ALT (IU/L) Median (min-max)	n	HBsAg (IU/ml) Median (min-max)	n	HBeAg (COI) Median (min-max)	n	Anti-HBc-IgM Positive/negative
1	0-19	4.181 ± 0.4521	27	31.95 (7.9-1000)	30	754.3 (0.04-52000)	30	0.21 (0.09-1522)	30	5/16
2	20-73	3.916 ± 0.3293	39	36.7 (4.7-2260)	58	131.8 (0.04-52000)	58	0.12 (0.088-1909)	56	12/15
3	74-502	3.900 ± 0.4456	21	27.15 (6.7-87.4)	24	219.3 (0.04-52000)	28	2.87 (0.106-1763)	24	5/11
Statistics		ANOVA		K-W		K-W		K-W		X^2^
*P*		0.862		0.284		0.071		0.029*		>0.05
*P* (M-W)	1 *vs* 2 1 *vs* 3 2 *vs* 3							0.937 0.374 0.023*		
*P* **^a^**		0.300		0.898		0.000***		0.834		0.756

ANOVA, One-way analysis of variance; K-W, Kruskal-Wallis test; M-W, Mann-Whitney test; X^2^, Chi-squared tests; COI, cut off index; COI=sample value/cut off value. COI>1.0 means positive result; COI<1.0 means negative result.

P^a^, Multivariate linear regression analysis; *p < 0.05, ***< 0.001.

## 4 Discussion

Although numerous studies have confirmed the important influences of host HBV-specific T cell immunity on the disease progression (5–7, 53), antiviral efficacy (8) and recurrence after therapy discontinuation (10, 12), the clinical uses of T cell epitope-based therapeutic vaccines and HBV-specific T cell detection are still limited. This is mainly contributed to the high diversity of HLA alleles and T cell epitopes, the key factors that must be taken in account when preparing an epitope vaccine or T cell detection kit. By now, only round 205 CD8^+^ T cell epitopes have been validated as summarized in our recent review. Of which, 121 (59.0%) epitopes are restricted by HLA-A0201, A2402 or B0702. The remainder are restricted mainly by 12 HLA-A, 5 HLA-B and 1 HLA-C supertypes (24). Obviously, the current library of validated T cell epitopes of HBV cannot cover the major populations in an indicated geographic region. More efforts are required to identify more T cell epitopes restricted to the regional prevalent HLA supertypes, especially for the HLA alleles prevalent in Asian populations with a high HBV incidence (54).

This study focuses on three points which different from previous studies: firstly, screening the HBV antigen epitopes presented by a series of high-frequency HLA-A allotypes in Asia. Each allotype has a gene frequency of >1% in Chinese and Northeast Asian populations. Totally, 13 kinds of predominant HLA-A allotypes were selected here and gather a total HLA-A allele frequency of around 95.5% in Chinese population while 94%, 83%, 80%, 70% and 63% in Northeast Asia, Southeast Asia, Europe, South America, and North America populations, respectively (http://www.allelefrequencies.net). The entire amino acid sequences of HBsAg, HBeAg, HBx and HBpol proteins from HBV C, B, A and D genotypes were screened for the CD8^+^ T cell epitopes restricted by the thirteen HLA-A allotypes, using the combined system of *in silico* prediction first and cellular functional validation later, an efficient, low-cost, and currently wide-used system for T cell epitope screening. Secondly, as known, one T cell epitope usually can be presented by several HLA allotypes with distinct binding affinity, named HLA cross-restriction. However, identifying the HLA restriction and cross-restriction of a T cell epitope is usually difficult due to the lack of standard methods. Therefore, most HBV T cell epitopes reported previously have not been elaborated for their HLA restriction, especially the cross-restriction by multiple HLA allotypes. Some epitopes have been elucidated only by *in silico* prediction or mere guesswork according to patient’s HLA serotypes or genotypes, such as HLA-A2 restricted epitopes (44, 55, 56), A24 (43), A3 (37, 47), A11 (37, 57) and A33 (41) restricted HBV epitopes. Thus this study not only validated the immunogenicity of 62 HBV T cell epitopes using *ex vivo* and *in vitro* cellular functional experiments, but also further identified their HLA-A cross-restrictions using multiple approaches. Thirdly, this study provided a library of HBV antigen CD8^+^ T cell epitopes that not only can target as many as 105 HBV-specific CD8^+^ T cell clones to reflecting patient’s T cell immune function, but also fits to the herd HLA genetic characteristics of Chinese and Northeast Asian populations, thus can be used to prepare ELISPOT or FluoroSpot kit and monitor HBV-specific T cell responses for broad patients.

In order to elucidate the HLA-A cross-restriction of 62 validated epitopes, the data from five approaches were taken into account comprehensively in this study as displayed in **Table 1**: *in silico* predicted HLA-A restriction by five prediction algorithms; *ex vivo* ELISPOT-positive patient’s HLA-A genotype; *in vitro* CD8^+^ T cells-activating patient’s HLA-A genotype; HLA/peptide docking and molecular dynamic simulation; and peptide competitive binding assay using HMy2.CIR cell lines expressing the indicated HLA-A allotype. Additionally, the PBMCs from patients carrying homologous HLA-A alleles were also used to identify the HLA-A restriction of partial epitopes by *ex vivo* ELISPOT assay, and 24 epitopes were confirmed to be presented by the indicated HLA-A allotype. Of note is that data from theoretical prediction were partially inconsistent with the ELISPOT-positive patients’ HLA-A genotypes. For example, P51 was supposed to be an HLA-A2402 restricted epitope as predicted, but it induced positive T cell responses in the PBMCs from an HLA-A0201^+^/A0207^+^ patient. Similar data for P77, 78, 95, 103, and 108 were also observed. These discordant results should be contributed by the inaccuracy of theoretical prediction, the small cohort of HLA-A matched patients, and the technical pitfall of ELISPOT assay. In our experiences, only around half of predicted epitopes can be validated finally as immunogenic epitopes in cellular functional experiments. In another hand, the real-world epitope can induce T cell response only in partial HLA-matched patients, but not in all HLA-matched patients, particularly in the *ex vivo* ELISPOT assay. Therefore, in this case, we further evaluated the binding affinity of the indicated epitopes with associated HLA-A allotypes, such as P51 with HLA-2402, A0201 and A0207, using HLA/peptide docking and molecular dynamic simulation, and peptide competitive binding assay. The prime-affinity calculated from bioinformatics analysis displayed the strongest binding of P51 with HLA-A2402 (-243.243), and slightly stronger binding with HLA-A0201 (-160.656) than with HLA-0207 (-142.36) (**Table S4**). In general, more negative the prime-affinity, stronger the bond. In parallel, peptide competitive binding showed the intermediate affinity of P51 with HLA-A2402 (39.97% and 52.50% inhibition at 5μM and 15μM, respectively), low affinity with HLA-A0207 (43.00% inhibition at 5μM or 15μM), and no affinity with HLA-A0201 (2.00% and 4.50% at 5μM and 15μM, respectively) (**Table 2**). We also can find partial discordant results between bioinformatics analysis and peptide competitive binding, such as P51, P103, P108, et al, but Pearson correlation tests confirmed the correlation coefficient of data from the two methods. Taken together, although not perfect, we provided the original theoretical and experimental data about the HLA-A allotypes associated with each in-house validated epitope and elucidated their HLA-A cross-restrictions to some extent.

Of note is that 13 of the 25 CD8^+^ T cell epitope peptides simultaneously induced CD4^+^ T cell responses in the 7 days of co-cultures of peptide with patient’s PBMCs, as detected by IFN-γ intracellular staining and flow cytometry (**Figure 2**). Similar results were also found in our previous study about T cell epitopes screening of SARS-CoV-2, in which 44 (36.66%) of the 120 validated CD8^+^ T cell epitope peptides simultaneously elicited CD4^+^ T cells with a two- to six-fold increase in IFN-γ^+^/CD3^+^/CD8^-^ T cell frequency in the 14 days of DC-peptide-PBL cocultures. However, after immunization with the 31 validated CD8^+^ T cell epitope peptides cocktail, only very weak CD4^+^ T cell responses were found in the HLA-A2/DR1 transgenic mice, as detected by IFN-γ intracellular staining (50). These data may suggest that a high concentration of short epitope peptides (9- or 10-mer, 20 μg/mL) may also be presented to CD4^+^ T cells by HLA class II molecules onto DC and B cells in the 7-14 days of coculture system but not under *in vivo* conditions. The underlying mechanism remains to be further elucidated. In this study, the 62 T cell epitopes presented by HLA-A allotypes were validated by *ex vivo* IFN-γ ELISPOT assay using patient’s PBMCs. Of which, whether some epitope peptides also stimulate CD4^+^ T cell to producing IFN-γ in the 20 hours of ELISPOT assay remains unclear.

The clinical detection of host HBV-specific T cells is still very limited, since no standard T cell epitope library covering broad patients is available thus far. Most studies have utilized panels of overlapping peptides (OLPs) spanning overall HBV antigens (peptide scanning) rather than functionally validated epitope peptides (VEPs) in the ELISPOT or FluoroSpot assay for HBV-specific T cell enumeration or in the mass cytometry for T cell functional phenotype analysis, after *ex vivo* co-culture of OLPs pools with host lymphocytes (12, 58–61). Only few researchers used a small amount of VEPs for the patient cohort with matching HLA allotype (62). As known, OLPs are not real-world proven T cell epitopes, most of them are pseudo-epitopes with unknown HLA restrictions, thus should not be a standard epitope library. More importantly, due to the large amount of OLPs spanning overall HBV polyproteins, peptide scanning is a high-cost and laborious method. For CD8^+^ T cell epitope, HBsAg, HBeAg, HBx and HBpol contain 131, 68, 49, and 279 OLPs (9-mer), respectively, when overlapping 6 amino acids.

This study makes efforts to explore a potent alternative way to detect HBV-specific T cells. We set a peptide library of 105 VEPs which cover most prevalent HLA-A allotypes and majority patients in Chinese and Northeast Asian populations. Of course, the current HLA-A restricted VEPs library cannot decipher global CD8^+^ T cell responses against HBV infection. We will further screen the epitopes presented by prevalent HLA-B and C allotypes to enrich the VEPs libraries for broader representation of target T cell clones. So currently we cannot perform the methodological comparison with the OLPs-used ELISPOT assay, although OLPs also can not define host global T cell responses. Whether using VEPs will provide more information than the use of OLPs remains to be demonstrated later. But the initial data from IFN-γ ELISPOT assay using the 105 HLA-A restricted VEPs displayed significant differences in the stratification analysis of HBV-specific T cell responses for 116 HBV infected patients. Multivariate linear regression analysis confirmed the significant correlation between HBsAg levels and SFUs levels. These conclusions mostly are consistent with previous researches using OLPs (19, 63–66). To get the more convincing and ample evidences, we will further enrich the VEPs library and expand the patient cohort to decipher the correlations of HBV-specific T cell responses with clinical and laboratory features.

Taken together, this study functionally validated 62 HBV CD8^+^ T cell epitopes and identified their cross-restriction by twelve prevalent HLA-A allotypes using multiple approaches. A peptide library of 105 VEPs covering the herd polymorphism of HLA allotypes in Chinese and Northeast Asian populations were achieved and followed by the evaluation of HBV-specific T cell responses for HBV infected patients using ELISPOT assay. Overall, this study provides novel HBV CD8^+^ T cell epitopes for the design and development of therapeutic vaccines inducing antiviral CD8^+^ T cell responses, and also provides an alternative way beyond OLPs for clinical test of HBV-specific T cell response.

## Data Availability Statement

The datasets presented in this study can be found in online repositories. The names of the repository/repositories and accession number(s) can be found in the article/**Supplementary Material**.

## Ethics Statement

The studies involving human participants were reviewed and approved by Clinical Ethics Committee of Nanjing Second Hospital. The patients/participants provided their written informed consent to participate in this study.

## Author Contributions

JL, JQ, and CS designed and supervised the research. YD and ZZ performed the main experiments of this study. XYL and XM performed the bioinformatics analysis. CZ and XJ assisted in the *in silico* prediction of T cell epitopes and set up the functional validation experiments of candidate epitopes using patient’s PBMCs and IFN-γ ELISPOT assay. XTL collected patients’ blood samples, prepared PBMCs and performed HLA-A genotyping. YW assisted in the generation of HMy2.1CIR cell lines expressing indicated HLA-A allotypes and reviewed the HBV T cell epitopes reported previously. CS and YD organized the whole data and wrote the manuscript with discussions from all authors. All authors contributed to the article and approved the submitted version.

## Funding

This work was supported by Jiangsu Provincial Science and Technology Fund of China (BE2017714), Nanjing Municipal Hygiene and Health Fund of Jiangsu Province (zkx18043), and Jiangsu Provincial Hygiene and Health Fund (M2020088) as well as National Natural Science Foundation of China (31871322, 82041006). The sponsors had no role in study design, data collection and analysis, preparation of the manuscript, or decision to submit the article for publication.

## Conflict of Interest

The authors declare that the research was conducted in the absence of any commercial or financial relationships that could be construed as a potential conflict of interest.

## Publisher’s Note

All claims expressed in this article are solely those of the authors and do not necessarily represent those of their affiliated organizations, or those of the publisher, the editors and the reviewers. Any product that may be evaluated in this article, or claim that may be made by its manufacturer, is not guaranteed or endorsed by the publisher.

## Supplementary Material

Supplementary Figure 1 Homologous analysis of HBsAg, HBeAg, HBx and HBpol proteins from HBV C, A, B, and D genotypes. The entire amino acid sequences of each protein from different genotypes of HBV were obtained from UniProt database, aligned and used for *in silico* prediction of HBV antigen T cell epitopes presented by HLA-A allotypes.

Supplementary Figure 2 The validated HBV epitope peptides induced CD8^+^ T cell responses in the *in vitro* cocultures with patients’ PBMCs. The PBMCs were stimulated with the indicated epitope peptides for seven days *in vitro* and followed by IFN-γ intracellular staining and flow cytometry. Totally, 25 epitope peptides were tested using the PBMCs from 19 patients with matching HLA-A alleles. **(A)** The flow plots of IFN-γ^+^ cells in CD3^+^/CD8^+^ T cell population in each coculture. **(B)** The flow plots of IFN-γ^+^ cells in CD3^+^/CD8^-^ T cell population in each coculture.

Supplementary Figure 3 Consensus sequences of HBsAg, HBeAg, HBx and HBpol in different HBV genotypes. A huge number of sequences of HBsAg, HBeAg, HBx and HBpol in HBV genotype A, B, C and D were collected from HBVdb database. Multiple sequence alignments were performed and analyzed to obtain the conservative regions of these sequences in a genotype-dependent way. The conservative properties of each amino acid in each protein were judged by the threshold of 100%, 95%, 80%, and the amino acids were highlighted in different colors (100%: red, ≥95%: yellow, ≥80%: black in gray background, and <80%: black in white background).

Supplementary Figure 4 The binding pattern between the main anchor residues of HBV epitope peptides with HLA-A molecules. The conformational interaction between P2, PΩ and the whole length of HBV epitope peptide (FLWEWASVR), (MMWYWGPSL) and (STLPETTVVR) with HLA-A0201 (PDB ID: 5F9J), A2402 (PDB ID: 2BCK) and A1101 (PDB ID: 5WKF), respectively, were displayed in 3D structures of peptide-binding groove of HLA-A molecules.

Supplementary Figure 5 Hmy2.CIR cell lines expressing indicated HLA-A allotypes. The transfected Hmy2.1 CIR cell lines expressing HLA-A2402, A0207, A0201, A0203, A0206, A0101, A1101, A3101, A3303, A3001, A1102 or A0301 were generated, respectively, and stained with FITC-anti-HLA-A24, PE-anti-HLA-A2 or PE-anti-HLA-ABC, then sorted by flow cytometry. The purity of these transfected HMy2.CIR cell lines was 80% to 94% after sorting.

Supplementary Figure 6 Binding affinity analysis of 62 validated HBV epitope peptides with corresponding HLA-A allotypes using peptide competitive binding assay. A series of unlabeled HBV epitope peptides were co-incubated with fluorescently labeled reference peptides and transfected HMy2.CIR cell lines expressing the indicated HLA-A allotypes. Then the competitively binding (%) of each HBV epitope peptide at 5μM and 15μM was calculated by measuring the percentage of fluorescence-positive HMy2.CIR cells. Blue line and purple line represented the line diagram of cell fluorescence strength under 5μM and 15μM HBV epitope peptide, respectively. Gray filled and red line represented the cell maximal fluorescence (cells with FITC-labeled reference peptide) and no peptide control (cell background fluorescence with 1640 medium alone), respectively.

## Abbreviations

HBV, Hepatitis B Virus; LC, Liver cirrhosis; HCC, Hepatocellular carcinoma; CHB, Chronic Hepatitis B; CTL, Cytotoxic T lymphocytes; HLA, Human leukocyte antigens; ELISPOT, Enzyme-linked immunosorbent spot; PBMCs, Peripheral blood mononuclear cells; ALT, Alanine aminotransferase; PHA, Phytohemagglutinin; SFU, Spot forming units; PDB, Worldwide Protein Data Bank; MM-GBSA, Generalized Bonn surface area based on molecular mechanics; OLPs, Overlapping peptides; VEPs, Validated epitope peptides.

## References

[1] World Health Organization. Global Hepatitis Report 2017. Geneva, Switzerland: World Health Organization (2017).

[2] Liu J Liang W Jing W Liu M. Countdown to 2030: Eliminating Hepatitis B Disease, China. Bull World Health Organ (2019) 97(3):230–8. doi: 10.2471/BLT.18.219469 PMC6453311 30992636

[3] Mohd-Ismail NK Lim Z Gunaratne J Tan YJ. Mapping the Interactions of HBV cccDNA With Host Factors. Int J Mol Sci (2019) 20(17):4276. doi: 10.3390/ijms20174276 PMC6747236 31480501

[4] Isogawa M Tanaka Y. Immunobiology of Hepatitis B Virus Infection. Hepatol Res (2015) 45(2):179–89. doi: 10.1111/hepr.12439 25331910

[5] Tseng T Huang L. Immunopathogenesis of Hepatitis B Virus. J Infect Dis (2017) 216(suppl_8):S765–70. doi: 10.1093/infdis/jix356 29156047

[6] Cheng Y Zhu YO Becht E Aw P Chen J Poidinger M. Multifactorial Heterogeneity of Virus-Specific T Cells and Association With the Progression of Human Chronic Hepatitis B Infection. Sci Immunol (2019) 4(32):eaau6905. doi: 10.1126/sciimmunol.aau6905 30737354

[7] Wang X He Q Shen H Lu X Sun B. Genetic and Phenotypic Difference in CD8(+) T Cell Exhaustion Between Chronic Hepatitis B Infection and Hepatocellular Carcinoma. J Med Genet (2019) 56(1):18–21. doi: 10.1136/jmedgenet-2018-105267 29666149 PMC6327916

[8] Xia Y Liang TJ. Development of Direct-Acting Antiviral and Host-Targeting Agents for Treatment of Hepatitis B Virus Infection. Gastroenterology (2019) 156(2SI):311–24. doi: 10.1053/j.gastro.2018.07.057 PMC6340783 30243618

[9] Tang TJ Kwekkeboom J Mancham S Binda RS de Man RA Schalm SW. Intrahepatic CD8(+) T-Lymphocyte Response Is Important for Therapy-Induced Viral Clearance in Chronic Hepatitis B Infection. J Hepatol (2005) 43(1):45–52. doi: 10.1016/j.jhep.2005.01.038 15893402

[10] Papatheodoridis G Vlachogiannakos I Cholongitas E Wursthorn K Thomadakis C Touloumi G. Discontinuation of Oral Antivirals in Chronic Hepatitis B: A Systematic Review. Hepatology (2016) 63(5):1481–92. doi: 10.1002/hep.28438 27100145

[11] EASL. 2017 Clinical Practice Guidelines on the Management of Hepatitis B Virus Infection. J Hepatol (2017) 67(2):370–98. doi: 10.1016/j.jhep.2017.03.021 28427875

[12] Rivino L Le Bert N Gill US Kunasegaran K Cheng Y Tan DZM. Hepatitis B Virus-Specific T Cells Associate With Viral Control Upon Nucleos(T)Ide-Analogue Therapy Discontinuation. J Clin Invest (2018) 128(2):668–81. doi: 10.1172/JCI92812 PMC5785266 29309050

[13] Elahi S Horton H. Association of HLA-Alleles With the Immune Regulation of Chronic Viral Infections. Int J Biochem Cell B (2012) 44(8):1361–5. doi: 10.1016/j.biocel.2012.05.003 PMC3380151 22595281

[14] Wang L Zou Z Wang K. Clinical Relevance of HLA Gene Variants in HBV Infection. J Immunol Res (2016) 2016:9069375. doi: 10.1155/2016/9069375 27243039 PMC4875979

[15] Lumley S Noble H Hadley MJ Callow L Malik A Chua YY. Hepitopes: A Live Interactive Database of HLA Class I Epitopes in Hepatitis B Virus. Wellcome Open Res (2016) 1:9. doi: 10.12688/wellcomeopenres.9952.1 27976751 PMC5142601

[16] Zheng J Ou Z Lin X Wang L Liu Y Jin S. Analysis of Epitope-Based Vaccine Candidates Against the E Antigen of the Hepatitis B Virus Based on the B Genotype Sequence: An *In Silico* and *In Vitro* Approach. Cell Immunol (2018) 329:56–65. doi: 10.1016/j.cellimm.2018.04.015 29724463

[17] Yamamiya D Mizukoshi E Kaji K Terashima T Kitahara M Yamashita T. Immune Responses of Human T Lymphocytes to Novel Hepatitis B Virus-Derived Peptides. PloS One (2018) 13(6):e198264. doi: 10.1371/journal.pone.0198264 PMC5983448 29856876

[18] Zhang Y Wu Y Deng M Xu D Li X Xu Z. CD8(+) T-Cell Response-Associated Evolution of Hepatitis B Virus Core Protein and Disease Progress. J Virol (2018) 92(17):e02120–17. doi: 10.1128/JVI.02120-17 PMC6096822 29950410

[19] Hoogeveen RC Robidoux MP Schwarz T Heydmann L Cheney JA Kvistad D. Phenotype and Function of HBV-Specific T Cells Is Determined by the Targeted Epitope in Addition to the Stage of Infection. Gut (2019) 68(5):893–904. doi: 10.1136/gutjnl-2018-316644 30580250

[20] Tham C Kah J Tan AT Volz T Chia A Giersch K. Hepatitis Delta Virus Acts as an Immunogenic Adjuvant in Hepatitis B Virus-Infected Hepatocytes. Cell Rep Med (2020) 1(4):100060. doi: 10.1016/j.xcrm.2020.100060 33205065 PMC7659593

[21] Liu Q Tian Y Li Y Zhang W Cai W Liu Y. *In Vivo* Therapeutic Effects of Affinity-Improved-TCR Engineered T-Cells on HBV-Related Hepatocellular Carcinoma. J Immunother Cancer (2020) 8(2):e1748. doi: 10.1136/jitc-2020-001748 PMC7745518 33323464

[22] Wang H Luo H Wan X Fu X Mao Q Xiang X. TNF-Alpha/IFN-Gamma Profile of HBV-Specific CD4 T Cells Is Associated With Liver Damage and Viral Clearance in Chronic HBV Infection. J Hepatol (2020) 72(1):45–56. doi: 10.1016/j.jhep.2019.08.024 31499130

[23] de Beijer MTA Jansen DTSL Dou Y van Esch WJE Mok JY Maas MJP. Discovery and Selection of Hepatitis B Virus-Derived T Cell Epitopes for Global Immunotherapy Based on Viral Indispensability, Conservation, and HLA-Binding Strength. J Virol (2020) 94(7):e01663–19. doi: 10.1128/JVI.01663-19 PMC7081907 31852786

[24] Wu Y Ding Y Shen C. A Systematic Review of T Cell Epitopes Defined From the Proteome of Hepatitis B Virus. Vaccines (2022) 10(2):257. doi: 10.3390/vaccines10020257 35214714 PMC8878595

[25] Voorter CE Palusci F Tilanus MG. Sequence-Based Typing of HLA: An Improved Group-Specific Full-Length Gene Sequencing Approach. Methods Mol Biol (2014) 1109:101–14. doi: 10.1007/978-1-4614-9437-9_7 24473781

[26] Burley SK Bhikadiya C Bi C Bittrich S Chen L Crichlow GV. RCSB Protein Data Bank: Powerful New Tools for Exploring 3D Structures of Biological Macromolecules for Basic and Applied Research and Education in Fundamental Biology, Biomedicine, Biotechnology, Bioengineering and Energy Sciences. Nucleic Acids Res (2021) 49(D1):D437–51. doi: 10.1093/nar/gkaa1038 PMC7779003 33211854

[27] Kong R Wang F Zhang J Wang F Chang S. CoDockPP: A Multistage Approach for Global and Site-Specific Protein-Protein Docking. J Chem Inf Model (2019) 59(8):3556–64. doi: 10.1021/acs.jcim.9b00445 31276391

[28] Zhu K Day T Warshaviak D Murrett C Friesner R Pearlman D. Antibody Structure Determination Using a Combination of Homology Modeling, Energy-Based Refinement, and Loop Prediction. Proteins (2014) 82(8):1646–55. doi: 10.1002/prot.24551 PMC5282925 24619874

[29] Beard H Cholleti A Pearlman D Sherman W Loving KA. Applying Physics-Based Scoring to Calculate Free Energies of Binding for Single Amino Acid Mutations in Protein-Protein Complexes. PloS One (2013) 8(12):e82849. doi: 10.1371/journal.pone.0082849 24340062 PMC3858304

[30] Kessler JH Benckhuijsen WE Mutis T Melief CJ van der Burg SH Drijfhout JW. Competition-Based Cellular Peptide Binding Assay for HLA Class I. Curr Protoc Immunol (2004) Chapter 18:12–8. doi: 10.1002/0471142735.im1812s61 18432926

[31] Tsai SL Chen MH Yeh CT Chu CM Lin AN Chiou FH. Purification and Characterization of a Naturally Processed Hepatitis B Virus Peptide Recognized by CD8+ Cytotoxic T Lymphocytes. J Clin Invest (1996) 97(2):577–84. doi: 10.1172/JCI118450 PMC507052 8567982

[32] Sobao Y Sugi K Tomiyama H Saito S Fujiyama S Morimoto M. Identification of Hepatitis B Virus-Specific CTL Epitopes Presented by HLA-A*2402, the Most Common HLA Class I Allele in East Asia. J Hepatol (2001) 34(6):922–9. doi: 10.1016/s0168-8278(01)00048-4 11451178

[33] Guillaume P Picaud S Baumgaertner P Montandon N Schmidt J Speiser DE. The C-Terminal Extension Landscape of Naturally Presented HLA-I Ligands. Proc Nat Acad Sci (2018) 115(20):5083–8. doi: 10.1073/pnas.1717277115 PMC5960288 29712860

[34] Zhou M Xu D Li X Li H Shan M Tang J. Screening and Identification of Severe Acute Respiratory Syndrome-Associated Coronavirus-Specific CTL Epitopes. J Immunol (2006) 177(4):2138–45. doi: 10.4049/jimmunol.177.4.2138 16887973

[35] Tsuboi A Oka Y Udaka K Murakami M Masuda T Nakano A. Enhanced Induction of Human WT1-Specific Cytotoxic T Lymphocytes With a 9-Mer WT1 Peptide Modified at HLA-A*2402-Binding Residues. Cancer Immunol Immunother (2002) 51(11-12):614–20. doi: 10.1007/s00262-002-0328-9 PMC11032939 12439606

[36] Bengsch B Martin B Thimme R. Restoration of HBV-Specific CD8+ T Cell Function by PD-1 Blockade in Inactive Carrier Patients Is Linked to T Cell Differentiation. J Hepatol (2014) 61(6):1212–9. doi: 10.1016/j.jhep.2014.07.005 25016223

[37] Bertoni R Sidney J Fowler P Chesnut RW Chisari FV Sette A. Human Histocompatibility Leukocyte Antigen-Binding Supermotifs Predict Broadly Cross-Reactive Cytotoxic T Lymphocyte Responses in Patients With Acute Hepatitis. J Clin Invest (1997) 100(3):503–13. doi: 10.1172/JCI119559 PMC508216 9239396

[38] Nayersina R Fowler P Guilhot S Missale G Cerny A Schlicht HJ. HLA A2 Restricted Cytotoxic T Lymphocyte Responses to Multiple Hepatitis B Surface Antigen Epitopes During Hepatitis B Virus Infection. J Immunol (1993) 150(10):4659–71. 7683326

[39] Rehermann B Fowler P Sidney J Person J Redeker A Brown M. The Cytotoxic T Lymphocyte Response to Multiple Hepatitis B Virus Polymerase Epitopes During and After Acute Viral Hepatitis. J Exp Med (1995) 181(3):1047–58. doi: 10.1084/jem.181.3.1047 PMC2191941 7532675

[40] Li J Han Y Jin K Wan Y Wang S Liu B. Dynamic Changes of Cytotoxic T Lymphocytes (CTLs), Natural Killer (NK) Cells, and Natural Killer T (NKT) Cells in Patients With Acute Hepatitis B Infection. Virol J (2011) 2(8):199. doi: 10.1186/1743-422X-8-199 PMC3096949 21535873

[41] Pan X Ding H Zhou X Tien P. Identification of Hepatitis B Virus-Specific CTL Epitopes Presented by HLA-A*33:03 in Peripheral Blood Mononuclear Cells From Patients and Transgenic Mice. Biochem Biophys Res Commun (2014) 449(1):135–40. doi: 10.1016/j.bbrc.2014.05.001 24813999

[42] Logean A Rognan D. Recovery of Known T-Cell Epitopes by Computational Scanning of a Viral Genome. J Comput Aided Mol Des (2002) 16(4):229–43. doi: 10.1023/a:1020244329512 12400854

[43] Kondo Y Asabe S Kobayashi K Shiina M Niitsuma H Ueno Y. Recovery of Functional Cytotoxic T Lymphocytes During Lamivudine Therapy by Acquiring Multi-Specificity. J Med Virol (2004) 74(3):425–33. doi: 10.1002/jmv.20194 15368520

[44] Urbani S Boni C Amadei B Fisicaro P Cerioni S Valli MA. Acute Phase HBV-Specific T Cell Responses Associated With HBV Persistence After HBV/HCV Coinfection. Hepatology (2005) 41(4):826–31. doi: 10.1002/hep.20614 15726541

[45] Li X Liu Y Xu Z Wan Z Bai S Mao P. A Complete Genomic Analysis of Hepatitis B Virus Isolated From 516 Chinese Patients With Different Clinical Manifestations. J Med Virol (2013) 85(10):1698–704. doi: 10.1002/jmv.23640 23852705

[46] Depla E van der Aa A Livingston BD Crimi C Allosery K De Brabandere V. Rational Design of a Multiepitope Vaccine Encoding T-Lymphocyte Epitopes for Treatment of Chronic Hepatitis B Virus Infections. J Virol (2008) 82(1):435–50. doi: 10.1128/JVI.01505-07 PMC2224390 17942551

[47] Mizukoshi E Sidney J Livingston B Ghany M Hoofnagle JH Sette A. Cellular Immune Responses to the Hepatitis B Virus Polymerase. J Immunol (2004) 173(9):5863–71. doi: 10.4049/jimmunol.173.9.5863 15494540

[48] Hwang YK Kim NK Park JM Lee K Han WK Kim HI. HLA-A2 1 Restricted Peptides From the HBx Antigen Induce Specific CTL Responses *In Vitro* and *In Vivo* . Vaccine (2002) 20(31-32):3770–7. doi: 10.1016/s0264-410x(02)00297-9 12399208

[49] Ding FX Wang F Lu YM Li K Wang KH He XW. Multiepitope Peptide-Loaded Virus-Like Particles as a Vaccine Against Hepatitis B Virus-Related Hepatocellular Carcinoma. Hepatology (2009) 49(5):1492–502. doi: 10.1002/hep.22816 19206147

[50] Jin X Ding Y Sun S Wang X Zhou Z Liu X. Screening HLA-A-Restricted T Cell Epitopes of SARS-CoV-2 and the Induction of CD8(+) T Cell Responses in HLA-A Transgenic Mice. Cell Mol Immunol (2021) 18(12):2588–608. doi: 10.1038/s41423-021-00784-8 PMC8561351 34728796

[51] Hayer J Jadeau F Deleage G Kay A Zoulim F Combet C. HBVdb: A Knowledge Database for Hepatitis B Virus. Nucleic Acids Res (2013) 41(Database issue):D566–70. doi: 10.1093/nar/gks1022 PMC3531116 23125365

[52] Kumar S Stecher G Tamura K. MEGA7: Molecular Evolutionary Genetics Analysis Version 7.0 for Bigger Datasets. Mol Biol Evol (2016) 33(7):1870–4. doi: 10.1093/molbev/msw054 PMC8210823 27004904

[53] Brinck-Jensen N Vorup-Jensen T Leutscher PDC Erikstrup C Petersen E. Immunogenicity of Twenty Peptides Representing Epitopes of the Hepatitis B Core and Surface Antigens by IFN-γ Response in Chronic and Resolved HBV. BMC Immunol (2015) 16(1):65. doi: 10.1186/s12865-015-0127-7 26526193 PMC4630833

[54] Nayagam S Thursz M Sicuri E Conteh L Wiktor S Low-Beer D. Requirements for Global Elimination of Hepatitis B: A Modelling Study. Lancet Infect Dis (2016) 16(12):1399–408. doi: 10.1016/S1473-3099(16)30204-3 27638356

[55] Webster GJ Reignat S Brown D Ogg GS Jones L Seneviratne SL. Longitudinal Analysis of CD8+ T Cells Specific for Structural and Nonstructural Hepatitis B Virus Proteins in Patients With Chronic Hepatitis B: Implications for Immunotherapy. J Virol (2004) 78(11):5707–19. doi: 10.1128/JVI.78.11.5707-5719.2004 PMC415806 15140968

[56] Lin CL Tsai SL Lee TH Chien RN Liao SK Liaw YF. High Frequency of Functional Anti-YMDD and -Mutant Cytotoxic T Lymphocytes After *In Vitro* Expansion Correlates With Successful Response to Lamivudine Therapy for Chronic Hepatitis B. Gut (2005) 54(1):152–61. doi: 10.1136/gut.2003.032920 PMC1774356 15591521

[57] Oseroff C Sette A Wentworth P Celis E Maewal A Dahlberg C. Pools of Lipidated HTL-CTL Constructs Prime for Multiple HBV and HCV CTL Epitope Responses. Vaccine (1998) 16(8):823–33. doi: 10.1016/s0264-410x(97)00264-8 9627940

[58] Bihl FK Loggi E Chisholm JV Hewitt HS Henry LM Linde C. Simultaneous Assessment of Cytotoxic T Lymphocyte Responses Against Multiple Viral Infections by Combined Usage of Optimal Epitope Matrices, Anti-CD3 mAb T-Cell Expansion and “RecycleSpot”. J Transl Med (2005) 3(1):20. doi: 10.1186/1479-5876-3-20 15888204 PMC1164435

[59] Cassaniti I Calarota SA Adzasehoun KMG Chiesa A Comolli G Parea M. Memory T Cells Specific for HBV Enumerated by a Peptide-Based Cultured Enzyme-Linked Immunospot Assay in Healthy HBV-Vaccinated Subjects. Hum Vaccin Immunother (2016) 12(11):2927:33. doi: 10.1080/21645515.2016.1204500 27392260 PMC5137528

[60] Jacobi FJ Wild K Smits M Zoldan K Csernalabics B Flecken T. OX40 Stimulation and PD-L1 Blockade Synergistically Augment HBV-Specific CD4 T Cells in Patients With HBeAg-Negative Infection. J Hepatol (2019) 70(6):1103–13. doi: 10.1016/j.jhep.2019.02.016 30826436

[61] Chen C Jiang X Liu X Guo L Wang W Gu S. Identification of the Association Between HBcAg-Specific T Cell and Viral Control in Chronic HBV Infection Using a Cultured ELISPOT Assay. J Leukocyte Biol (2021) 109(2):455–65. doi: 10.1002/JLB.5MA0620-023RR 32620046

[62] Sung PS Park DJ Kim JH Han JW Lee EB Lee GW. *Ex Vivo* Detection and Characterization of Hepatitis B Virus-Specific CD8(+) T Cells in Patients Considered Immune Tolerant. Front Immunol (2019) 10:1319. doi: 10.3389/fimmu.2019.01319 31244857 PMC6563765

[63] Wang FS Zhang Z. Host Immunity Influences Disease Progression and Antiviral Efficacy in Humans Infected With Hepatitis B Virus. Expert Rev Gastroenterol Hepatol (2009) 3(5):499–512. doi: 10.1586/egh.09.50 19817672

[64] Bertoletti A Ferrari C. Innate and Adaptive Immune Responses in Chronic Hepatitis B Virus Infections: Towards Restoration of Immune Control of Viral Infection. Gut (2012) 61(12):1754–64. doi: 10.1136/gutjnl-2011-301073 22157327

[65] Guidotti LG Chisari FV. Noncytolytic Control of Viral Infections by the Innate and Adaptive Immune Response. Annu Rev Immunol (2001) 19:65–91. doi: 10.1146/annurev.immunol.19.1.65 11244031

[66] Cornberg M Wong VW Locarnini S Brunetto M Janssen H Chan HL. The Role of Quantitative Hepatitis B Surface Antigen Revisited. J Hepatol (2017) 66(2):398–411. doi: 10.1016/j.jhep.2016.08.009 27575311

