# Supplementary material for: Screening and Identification of HBV Epitopes Restricted by Multiple Prevalent HLA-A Allotypes
Source: Front Immunol. 2022 Apr 7;13:847105. doi: 10.3389/fimmu.2022.847105 (PMC9021956; doi:10.3389/fimmu.2022.847105)
Supplement: Supplementary Figure 1 — Homologous analysis of HBsAg, HBeAg, HBx and HBpol proteins from HBV C, A, B, and D genotypes. The entire amino acid sequences of each protein from different genotypes of HBV were obtained from UniProt database, aligned and used for in silico prediction of HBV antigen T cell epitopes presented by HLA-A allotypes. [file DataSheet_1.pdf]

Supplementary Tables and Figures of the manuscript entitled "Screening and identification of HBV epitopes restricted by multiple prevalent HLA-A allotypes", and authored by Yan Ding, et al.

Table S1 Primers used in this study

Primers	Sequence(5'-3')	Anneal site	Length
A1	GAAACSGCCTCTGYGGGGAGAAGCAA	HLA-A intron l:21-26	985bp
A3	TGTTGGTCCCAATTGTCTCCCCTC	HLA-A intron 3:66-89	
A2F	AGCCGCGCCKGGASGAGGGTC	Exon 2 intron 2: 99-119	270bp
A2R	GGCCCGTCCGTGGGGGATGAG	Exon 2 intron 2: 37-57	
A3F	GTTTCATTTTGRTTKAGGCCA	Exon 3 intron 3: 150-171	276bp
A3R	TGTGGGAGGCCAGCCCGGGAGA	Exon 3 intron 3: 41-66	
AF	tatagggagacccaagctggATGGCCGTCATGGCGCCC	HLA-A CDS	1098bp
AR	tttttgttcgggcccaagctTCACACTTTACAAGCTGTGAGAGACA		
P-1F	AGCTTGGGCCCGAACAAA	pcDNATM3.1	5500bp
P-1R	CCAGCTTGGGTCTCCCTATAGTG		

Note: CDS, Coding sequence.

Table S2 *In silico* predicted 124 HBV CD8^{+} T cell epitopes were synthesized for further validation

HLA-A allotype	Epitope name	Protein	Virus genotype	Serotype	start	end	Epitope sequence	SYFPEITHI	BIMAS	SVMHC		IEDB		NETMHC	EPIJEN
										SYFPEITHI	MHCPEP	ANN	SMM		
								Score> 20	Score > 10	Score ≥ 0.5		IC50 < 500 nM		Threshold< 2.0	IC50 < 500 nM
A0201	P6	HBsAg	A/B/C/D	adw/adr/ayw	262	270	LLCLIFLLV	25	831.216	0.17	1	204.82	30.63	1.7	0.87
	P7	HBsAg	A/B/C/D	adw/adr/ayw	346	354	WLSLLVPFV	26	4047.23	1	0.73	6.95	11.64	0.06	0.26
	P8	HBsAg	A/B/C/D	adw/adr/ayw	359	368	GLSPTVWLSV	26				16.49	61.16	0.25	188.36
	P1	HBeAg	AD		47	56	FLPSDFFPSV	24	2309.961	1	0.89	4.13	11.87	0.01	5.41
	P11	HBeAg	A/B/C/D		147	155	YLVSFGVWI	23	1003	0.38	1	38.14	53.02	0.25	0.09
	P31	HBeAg	BC		47	56	FLPSDFFPSI	24	2309.961	1	0.89	4.13	11.87	0.01	364.75
	P32	HBeAg	A/B/C/D		129	137	LLWFHISCL	26	693.27	0.33	1	21.49	23.94	0.3	6.5
	P9	HBpol	A/C/D		442	450	GLSRYVARL	28				89.06	106.93	1	0.87
	P10	HBpol	A/B/C/D		573	581	FLLSLGIHL	24	363.59		1	9.41	15.85	0.1	0.11
	P33	HBpol	B		743	751	KLIGTHNSV	28	243.43	0.88	0.92	41.52	65.33	0.6	1.34
	P34	HBpol	CA		743	751	KLIGTDNSV	28				38.71	60.55	0.5	0.5
	P12	HBx	D		115	123	CLFKDWEEL	23	65.841	0.06	0.47	153.91	152.09	1.4	2.18
	P35	HBx	A/B/C/D		92	100	VLHKRTLGL	25	36.3	0.23	1.2	467.19	405.61	3	2.84
	P36	HBx	A/B/C/D		133	141	VLGGCRHKL	26	36.3	0.7	0.45	1950.3	491.03	3.5	4.3
	P37	HBx	BA		102	110	AMSTTDLEA	17	3.59			403.25	39.29	2.5	2.67
A1101	P13	HBsAg	C	adr	306	315	SMYPSCCCTK	20				69.6	471.7	0.4	862.98
	P14	HBsAg	C	adr/ayw	158	167	ASPISSIFSR	22				37.46	146.5	0.25	1757.9
	P38	HBsAg	C	adr/ayw	159	167	SPISSIFSR	16	0.3		0.07	2738.5	284.64	0.04	7.13

	P39	HBsAg	B	adw	159	167	SAISSISSK	16			11.58	41.72	0.04	7.13
	P40	HBsAg	B	adw	190	198	QAGFFSLTK	15	0.4	0.07	60.4	135.93	0.5	20.7
	P41	HBsAg	CD	adr/ayw/adw	190	198	QAGFFLLTR	20	10		1636.8	887.77	3.5	1.97
	P3	HBeAg	BC		117	125	YVNTNMGLK	23	2	0.12	69.82	60.72	0.5	2.24
	P4	HBeAg	A/B/C/D		148	156	LVSFGVWIR	19	0.8		424.66	463.77	1.7	52.84
	P42	HBeAg	A/B/C/D		170	179	STLPETTVVR	28	0.3		86.32	129.67	0.6	/
	P43	HBeAg	A/B/C/D		133	141	HISCLTFGR	14	0.24		204.4	314.99	1.1	21.33
	P15	HBpol	D		118	127	KVTKYLPLDK	25	12		130.94	105.65	0.8	/
	P16	HBpol	CD		671	679	PTYKAFLCK	25	0.4		81.77	72.83	0.6	8.91
	P27	HBpol	A/B/D		47	55	NVSIPWTHK	20	2		224.51	360.83	1.2	3.33
	P44	HBpol	AB		487	495	YVSLMLLYK	24	4		11	16	0.03	6.97
	P45	HBpol	C		487	495	YVSLLLLYK	24			11.22	14.63	0.04	5.81
	P46	HBpol	C		389	397	VVDFSQFSR	18	0.8	0.06	110.19	208.11	1.3	6.14
	P17	HBx	AD		82	91	TVNAHQILPK	26	4		15.6	15.7	0.07	/
	P18	HBx	A/B/C/D		104	113	STTDLEAYFK	21	2		13.84	34.38	0.1	5
	P47	HBx	B		82	91	TVNAHGNLPK	26	4		15.6	15.7	0.12	/
	P48	HBx	C		82	91	TVNAHQVLPK	26			16.51	19.05	0.08	/
	P49	HBx	A/B/C/D		105	113	TTDLEAYFK	21	2		18.63	34.38	0.1	5
A2402	P19	HBsAg	CD	ayw	379	387	LYSILSPFL	20			203.41	428.18	0.4	1.279
	P20	HBsAg	ACD	adr/ayw/adw	258	266	LFILLLCLI	20		0.08	2069.3	692.84	1.9	1.499
	P50	HBsAg	A/B/C/D	adr/ayw/adw	247	256	RWMCLRRFII	11			33.77	85.06	0.2	0.961
	P51	HBsAg	A/B/C/D	adr/ayw/adw	364	373	VWLSVIWMM W	4			57.01	308.83	0.3	0.548
	P21	HBeAg	D		52	60	FFPSVRDLL	21			2044.1	2216.3	1.8	1.197

	P22	HBeAg	D		116	124	SYVNTNMGL	21		0.32	1537	1338	1.2	1.322
	P23	HBeAg	A/B/C/D		146	154	EYLVSFGVW	13	9		341.06	330.85	0.6	1.573
	P52	HBeAg	A/B/C/D		131	139	WFHISCLTF	15	10		190.65	301.74	0.4	1.282
	P53	HBeAg	ABC		116	124	SYVNVNMGL	21		0.32	1031.7	1149.3	1.2	1.404
	P24	HBpol	A/B/C/D		756	764	KYTSFPWLL	24	400	0.36	14.45	55.03	0.02	1.582
	P25	HBpol	D		115	123	FYPKVTKYL	24			1031	1149	0.4	1.483
	P55	HBpol	B		62	70	LYSSTVPCF	21	100		26.4	77.55	0.04	1.82
	P56	HBpol	C		62	70	LYSSTVPVF	21			18.2	78.64	0.02	1.692
	P57	HBpol	B		115	123	FYPNVTKYL	24		0.16	198.85	585.64	0.4	1.418
	P26	HBx	D		142	150	VCAPAPCNF	19	200		122.72	445.28	0.25	0.695
	P28	HBx	A/B/C/D		132	140	FVLGGCRHK			0.46	843.43	569.9	1.8	
A3101	P5	HBsAg	CD	ayw	335	343	FLWEWASAR				63.9	81.9		28.97
	P58	HBsAg	A/B/C/D	adr/ayw/adw	245	253	GYRWMCLRR		1.2		39.3	90.67	0.5	89.13
	P59	HBsAg	A/B/C/D	adr/ayw/adw	244	253	PGYRWMCLR				139.47	106.52	1.1	115.08
	P60	HBpol	A/B/C/D		188	196	RTPSPRRRR				34.12	30.44	0.4	54.7
	P61	HBpol	A/B/C/D		106	114	RLKLIMPAR		12		10.03	10.38	0.09	13.96
	P62	HBpol	BC		580	588	HLNPHKTKR				9.86	17.55	0.09	21.04
	P63	HBx	A/B/C/D		130	138	KVFVLGGCR		18		32.56	24.74	0.4	232.27
	P64	HBx	C		130	138	MVFVLGGCR				65.97	42.8		103.04
A0206	P2	HBsAg	CD	ayw	194	202	FLLTRILTI				23.51	29.01		25.7
	P66	HBeAg	A/B/C/D		1	9	MQLFHLCLI				4.4	4.34	0.04	162.93
	P67	HBeAg	A/B/C/D		47	55	FLPSDFFPS				6.8	26.95	0.09	
	P68	HBpol	A/B/C/D		770	778	WILRGTSFV				14.49	27.58	0.3	8.65
	P69	HBx	BC		4	12	RVCCQLDPA				52.69	245.78	0.9	390.84

	P70	HBx	BCD		7	15	CQLDPARDV	270.96	123.75	2.5	93.97
A0207	P71	HBsAg	B	adw	371	379	MMWFWGPSL	281.4	876.31	0.15	
	P72	HBsAg	ACD	adr/ayw/adw	371	379	MMWYWGPSL	6903	823.85		
	P73	HBsAg	A/B/C/D	adr/ayw/adw	349	358	LLVPFVQWFV	453.8	2687.8	0.01	
	P74	HBeAg	BC		88	97	ILCWGELMNL	1136.6	3064.5	1.3	
	P76	HBpol	A/B/C/D		524	532	FLLAQFTSA	481.1	2400		
A3303	P77	HBsAg	A/B/C/D	adr/ayw/adw	243	252	CPGYRWMCLR	127.9	182.79		
	P78	HBsAg	C	adr/ayw	335	343	FLWEWASVR	38.5	69.92		
	P79	HBsAg	B	adw	334	343	KYLWGWASVR	131.8	142.04		
	P80	HBeAg	A/B/C/D		77	85	CSPHHTALR	85.1	121.36		
	P81	HBpol	A/B/C/D		729	737	ELLAACFAR	30.7	22.64		
	P82	HBpol	A/B/C/D		1	9	MPLSYQHFR	28	30.94		
	P29	HBpol	A/B/C/D		504	513	YSHPIILGFR	40.1	25.3		
	P83	HBx	B/C/A		69	77	CALRFTSAR	21.6	19.41		
	P84	HBx	A/B/C/D		64	72	SSAGPCALR	31.7	58.28		
	P122	HBsAg	A		335	343	YLWEWASVR	73.65	52.46		
A1102	P101	HBsAg	B/C/A	adw/adr/ayw	306	315	SMFPSCCCTK	53.5	41.14		
	P102	HBsAg	A/C/D	adw/adr/ayw	189	198	LQAGFFLLTR	398.5	510.44		
	P103	HBsAg	B	adw	189	198	LQAGFFSLTK	29	26.25		
	P104	HBeAg	B/C/D		12	21	CSCPTVQASK	284.6	204.96		
	P105	HBpol	A/B/C/D		665	674	QAFTFSPTYK	11.4	784.45		
	P106	HBpol	A/B/C/D		150	159	TLWKAGILYK	18.9	776		
A3001	P85	HBsAg	A/B/C	adw/adr/ayw	341	349	SVRFSWLSL	5.65	36.73	0.03	

	P86	HBsAg	A/B/C/D	adw/adr/ayw	250	258	CLRRFIIFL		79.51	169.42	0.5	
	P87	HBeAg	A/B/C/D		160	168	AYRPPNAPI		7.31	75.33	0.05	
	P88	HBeAg	B/C		109	117	ASRELVVSY		87.79	192.74	0.5	
	P89	HBpol	B/C		737	745	RSRSGAKLI		10.35	47.63	0.08	
	P90	HBpol	C/D		737	745	RSRSGANIL		11.71	83.17	0.04	
	P91	HBpol	B/C/A		829	837	RVHFASPLH		11.28	79.61	0.08	
	P92	HBx	B/C		94	102	HKRTLGLSA		271.74	42.66	1	
	P93	HBx	A/B/C/D		128	136	RLKVFVLGG		102.88	153.1	0.5	
	P94	HBx	A/B/C		91	99	KVLHKRTLGL		362.53	274.77	1.5	
A0203	P95	HBsAg	A/B/C/D	adw/adr/ayw	271	280	LLDYQGMLPV		5.72	20.5	0.125	
	P96	HBeAg	A/B/C/D		168	177	ILSTLPETTV		13.75	29.05	0.4	
	P97	HBeAg	B/C/D		8	17	LIISCSCPTV		31.29	22.06	0.9	
	P98	HBpol	A/B/C/D		525	533	LLAQFTSAI		3.07	16.8	0.025	3.56
	P99	HBpol	A/B/C/D		524	533	FLLAQFTSAI		3.34	14.01	0.03	
	P100	HBx	A/B/C/D		52	60	HLSLRGLPV		20.45	59.42	0.6	1.67
A0301	P107	HBsAg	B	adw	371	380	MMWFWGPSL Y		16.06	32.52	0.05	
	P108	HBsAg	A/C/D	adw/adr/ayw	371	380	MMWYWGPSL Y		8.21	36.07	0.015	
	P109	HBeAg	B/C		117	125	YVNVNMGLK		180.57	194	0.7	32.66
	P30	HBeAg	A/B/C		125	133	KIRQLLWFH		993.33	1034.6	2	3.4
	P54	HBpol	B		156	164	ILYKRESTR	1.27	68.73	126.9	0.4	44.26
	P65	HBpol	C/D		656	664	PLYACIQSK	0.93	82.66	60.65	0.4	14.32
	P110	HBpol	A/B/C/D		492	500	LLYKTYGRK		8.19	24.14	0.02	6.52

	P75	HBx	A/B/C/D		132	140	FVLGGCRHK	0.46	843.43	569.9	1.8	
A0101	P123	HBsAg	A/B	adw	327	335	PSSWAFAKY		455.64	703.96	0.4	11.99
	P124	HBeAg	B/C		49	57	PSDFFPSIR		8570.2	1768.2	2.5	
	P111	HBeAg	BAD		59	67	LLDTASALY		5.47	154.72	0.01	0.11
	P112	HBpol	A/B/C/D		427	435	SLDVSAAFY		9.47	366.9	0.01	8.24
	P113	HBpol	A/B/C/D		808	816	PTTGRTSLY		218.64	255.59	0.25	0.85
	P114	HBx	A/B/C		102	118	AMSTTDLEAY		149.77	884.71	0.2	
A2601	P115	HBsAg	A/B/C	adw/adr/ayw	17	25	SVPNPLGFF		65.49	321.08	0.08	
	P116	HBsAg	A/B/C/D	adw/adr/ayw	67	75	FTPPHGGLL		535.92	710.58	0.4	
	P117	HBeAg	B/C/A		142	151	ETVLEYLVSF		13.47	589.78	0.03	
	P118	HBeAg	C		142	151	ETVLEYLVSV		419.55	3417.4	0.3	
	P119	HBpol	A/B/C/D		726	735	HTAELLAACF		20.87	51.54	0.04	
	P120	HBpol	A/B/C		789	798	DPSRGRLGLY		271.67	1353.6	0.2	
	P121	HBx	A/B/C/D		104	112	STTDLEAYF		707.89	725.45	0.4	

Table S3 Characteristics of 56 HBV infected patients whose PBMCs displayed positive responses in IFN-γ ELISPOT assay with epitope peptide pools

Patient ID	Gender	Age (years)	Diagnose	Therapy	HLA-A genotype	HBsAg (IU/mL)	HBc-IgM	Viral load (IU/mL)	ALT (IU/L)
HBV1	m	52	LC	NAs	A*02:01/11:36	2310	negative	1.40×10^{6}	35.1
HBV2	f	63	HCC	no	A*24:02	>250	positive	1.90×10^{7}	211
HBV3	m	71	CHB	NAs	A*02:01/32:01	97.08	negative	$<5.0\times10^{2}$	56.2
HBV4	m	40	CHB	no	A*02:06/11:01	15274	negative	3.90×10^{3}	41.2
HBV5	m	55	LC	NAs	A*02:01/02:06	399	negative	$<5.0\times10^{2}$	34.6
HBV6	m	60	CHB	NAs	A*11:01/24:02	32.82	n.d	$<5.0\times10^{2}$	59.9
HBV7	m	54	LC	NAs	A*24:02	192.27	n.d	3.10×10^{3}	44.6
HBV8	f	70	LC	NAs	A*11:01/30:01	>250	negative	5.00×10^{3}	15.5
HBV9	f	34	CHB	no	A*24:02/33:03	38157	positive	n.d	87
HBV10	m	62	CHB	NAs	A*02:07/24:02	1.2	positive	9.90×10^{2}	18
HBV11	f	72	CHB	NAs	A*02:06/30:01	51.25	positive	2.20×10^{5}	51.5
HBV12	m	58	LC	NAs	A*02:03/24:02	242	n.d	$<5.0\times10^{2}$	29
HBV13	m	28	CHB	no	A*11:01/24:02	2649	positive	2.10×10^{7}	111.8
HBV14	m	50	LC	NAs	A*24:02	6.43	negative	$<5.0\times10^{2}$	20.6
HBV15	m	48	CHB	NAs	A*11:01	36.24	negative	$<5.0\times10^{2}$	61.3
HBV16	m	22	CHB	no	A*02:03/11:01	697.7	positive	2.00×10^{6}	150.8
HBV17	m	56	CHB	NAs	A*02:01/33:03	96.96	negative	4.70×10^{5}	567
HBV18	m	48	LC	NAs	A*11:01/02:01	141.1	n.d	$<5.0\times10^{2}$	34.6
HBV19	m	47	CHB	no	A*24:02/02:07	186.1	positive	$<5.0\times10^{2}$	730

HBV20	m	49	LC	NAs	A*02:01	376	lowpositive	<5.0×10^2	24.6
HBV21	m	32	CHB	NAs	A*02:01/02:07	1544	n.d	1.10×10^4	60.1
HBV22	m	52	HCC	NAs	A*24:02	126.98	n.d	<5.0×10^2	6
HBV23	m	70	LC	NAs	A*02:07/30:01	142.2	n.d	<5.0×10^2	38.7
HBV24	m	43	LC	NAs	A*11:01/24:63	1247	negative	2.56×10^2	32.5
HBV25	m	65	HCC	NAs	A*02:01/24:02	758	negative	<5.0×10^2	33.8
HBV26	f	46	HCC	NAs	A*24:02/26:01	402	negative	<5.0×10^2	40.9
HBV27	m	64	LC	NAs	A*11:01/31:01	756	n.d	<5.0×10^2	10.9
HBV28	m	34	CHB	NAs	A*02:01	1.18	n.d	2.93×10^2	78.8
HBV29	m	27	CHB	no	A*01:01/02:06	670	negative	6.70×10^2	258.9
HBV30	m	68	HCC	NAs	A*24:02/33:15	118	negative	<5.0×10^2	14.2
HBV31	m	39	CHB	NAs	A*01:01/02:06	>52000	negative	9.80×10^8	516
HBV32	m	28	CHB	no	A*24:02/02:07	4096	negative	5.10×10^2	135.5
HBV33	m	36	CHB	NAs	A*24:02	5254	negative	<5.0×10^2	22.2
HBV34	f	56	CHB	no	A*02:06	581	negative	<5.0×10^2	827
HBV35	m	43	CHB	NAs	A*24:02	>52000	negative	5.10×10^7	879
HBV36	f	45	CHB	NAs	A*24:02/24:109	>52000	n.d	1.60×10^4	189
HBV37	m	64	LC	NAs	A*24:02/11:01	776	positive	<5.0×10^2	65.7
HBV38	m	62	LC	NAs	A*24:02/33:03	824	negative	<5.0×10^2	11.2
HBV39	m	70	CHB	NAs	A*24*02	2373	positive	6.30×10^3	265
HBV40	f	43	CHB	no	A*24:02/02:07	51151	negative	4.00×10^8	129
HBV41	m	62	LC	NAs	A*0201/26:01	311	positive	1.60×10^4	76.9
HBV42	m	56	HCC	NAs	A*24:02/02:07	108	n.d	<5.0×10^2	28.7
HBV43	m	35	LC	NAs	A*02:03/30:01	29.22	negative	<5.0×10^2	96.7

HBV44	m	49	CHB	NAs	A*2402	1238	negative	1.50×10^{7}	64.3
HBV45	m	39	LC	NAs	A*11:01/24:02	41.2	negative	n.d	23.4
HBV46	f	36	CHB	no	A*02:01	0.09	positive	5.65×10^{2}	1338
HBV47	f	56	LC	NAs	A*02:01/11:01	127	positive	1.80×10^{3}	24.4
HBV48	m	36	CHB	NAs	A*02:01/02:07	383	negative	$<5.0\times10^{2}$	25.7
HBV49	f	50	LC	NAs	A*02:01/24:02	558	n.d	$<5.0\times10^{2}$	10.3
HBV50	f	65	HCC	NAs	A*0201	105	n.d	$<5.0\times10^{2}$	48.8
HBV51	m	27	CHB	NAs	A*0203/0301	37537	n.d	9.20×10^{7}	235
HBV52	m	51	CHB	no	A*1101/0207	11226	positive	2.70×10^{7}	431
HBV53	f	42	CHB	no	n.d	11441	n.d	6.80×10^{7}	45.8
HBV54	f	71	HCC	NAs	n.d	1213	negative	$<5.0\times10^{2}$	58
HBV55	f	42	AHB	NAs	A*0206/0201	>52000	positive	2.30×10^{6}	828
HBV56	m	39	CHB	NAs	A*0206	>52000	positive	8.10×10^{6}	444

Note: **f**, Female; **m**, Male; **NAs**, Nucleos(t)ide (acid) analogues; **n.d**, not detection.

Table S4 Bioinformatic analysis for the binding affinity of 45 validated HBV epitope peptides with corresponding HLA-A allotypes

Epitope	HBV peptide	HLA	PDB ID	Bond Peptide	Resolution	Δ affinity (Kal/mol)	Prime Affinity (Kal/mol)	Binding Free Energy (Kal/mol)
P19	LYSILSPFL	2402	5HGH	RYPLTFGWCF	2.39 Å	14.01	-230.979	
	LYSILSPFL	0201	5F9J	YLSPIASPL	2.51 Å	29.88	-205.509	
P20	IFILLLCLI	2402	5WWU	LYKKLKREITF	2.79 Å	14.61	-228.759	
P38	SPISSIFSR	0201	5HHM	GILGLVFTL	2.5 Å	25.03	-214.158	
	SPISSIFSR	1101	5WKH	GTSGSPIINR	3.2Å	17.02	-207.058	
P39	SAISSISSK	1101	5WKH	GTSGSPIINR	3.2Å	28.51	-195.561	
	SAISSISSK	0203	3OX8	FLPSDFFPSV	2.16 Å	57.62	-184.133	
P40	QAGFFSLTK	1101	5WKH	GTSGSPIINR	3.2Å	22.94	-201.319	
	QAGFFSLTK	3101	5WJL(Template)	GTSGSPIVNR	3.15Å	30.97	-194.895	
P41	QAGFFLLTR	1101	1Q94	AIFQSSMTK	2.4Å	-0.17	-202.268	
	QAGFFLLTR	1102	5WJL(Template)	GTSGSPIVNR	3.15Å	25.23	-204.629	
P51	VWLSVIWMMW	2402	5WXD	LYKKLKREMTF	3.3 Å	-9.87	-243.243	
	VWLSVIWMMW	0201	3I6K	TLACFVLAAV	2.8 Å	39.08	-160.656	
	VWLSVIWMMW	0207	3OXS	FLPSDFFPSV	1.75 Å	96.17	-142.36	
P71	MMWFWGPSL	2402	3QZW	RYPLTFGWCF	2.8 Å	30.74	-224.833	
	MMWFWGPSL	0207	3OXS	FLPSDFFPSV	1.75 Å	29.83	-209.52	
	MMWFWGPSL	0301	6O9C	TTAPFLSGK	2.45 Å	18.96	-186.072	
P72	MMWYWGPSL	0201	4WUU	RMFPNAPYL	3.05 Å	29.53	-232.845	
	MMWYWGPSL	0207	3OXS	FLPSDFFPSV	1.75 Å	21.85	-217.501	
	MMWYWGPSL	2402	2BCK	VYGFVRACL	2.8 Å	1.29	-203.137	
P77	CPGYRWMCLR	3303	5WJL(Template)	GTSGSPIVNR	3.15Å	-2.28	-219.146	
P78	FLWEWASVR	0201	5F9J	YLSPIASPL	2.51 Å	-10.79	-245.795	
	FLWEWASVR	3303	5WJL(Template)	GTSGSPIVNR	3.15Å	-19.43	-244.411	
	FLWEWASVR	2402	3QZW	RYPLTFGWCF	2.8 Å	33.28	-222.293	
P95	LLDYQGMLPV	203	3OX8	FLPSDFFPSV	2.16 Å	36.06	-206.186	
	LLDYQGMLPV	2402	4F7M	LYASPQLEGF	2.4 Å	20.23	-189.639	
P103	LQAGFFSLTK	0207	3OXS	FLPSDFFPSV	1.75 Å	58.05	-181.332	
	LQAGFFSLTK	2402	4F7M	LYASPQLEGF	2.4 Å	31.7	-178.245	
	LQAGFFSLTK	1102	5WJL(Template)	GTSGSPIVNR	3.15Å	65.2	-155.892	

P108	MMWYWGPSLY	2402	5HGD	RFPLTFGWCF	2.07 Å	16.42	-227.601	
	MMWYWGPSLY	0301	3RL2	QVPLRPMTYK	2.39 Å	7.96	-212.347	
	MMWYWGPSLY	0207	3OXS	FLPSDFFPSV	1.75 Å	39.17	-200.201	
P31	FLPSDFFPSI	0207	3OXS	FLPSDFFPSV	1.75 Å	-3.52	-234.402	
	FLPSDFFPSI	0201	5FDW	YLSPIASPLL	2.7 Å	6.22	-210.992	-91.356
	FLPSDFFPSI	1101	5GRD	SSCSSCPLSK	1.8 Å	/	/	-81.106
P42	STLPETTVVR	1101	5WKF	GTSGSPIVNR	2.95Å	23.62	-232.694	
	STLPETTVVR	0207	3OXS	FLPSDFFPSV	1.75 Å	50.86	-188.512	
P52	WFHISCLTF (HIE)	2402	3I6L	QFKDNVILL	2.4 Å	-16.95	-229.136	
	WFHISCLTF (HIE)	0207	3OXS	FLPSDFFPSV	1.75 Å	25.2	-214.149	
	WFHISCLTF (HIE)	0201	5SWQ	CVNGSCFTV	2.0 Å	1.38	-194.168	
	WFHISCLTF (HIP)	2402	3I6L	QFKDNVILL	2.4 Å	-12.31	-224.493	
	WFHISCLTF (HIP)	0207	3OXS	FLPSDFFPSV	1.75 Å	25.2	-219.55	
	WFHISCLTF (HIP)	0201	5SWQ	CVNGSCFTV	2.0 Å	-7.94	-203.491	
	WFHISCLTF (HID)	0207	3OXS	FLPSDFFPSV	1.75 Å	24.87	-214.484	
	WFHISCLTF (HID)	2402	3QZW	RYPLTFGWCF	2.8 Å	41.34	-214.233	
	WFHISCLTF (HID)	201	1DUZ	LLFGYPVYV	1.8 Å	9.25	-205.066	
P53	SYVNVNMGL	2402	3QZW	RYPLTFGWCF	2.8 Å	28.48	-230.186	
	SYVNVNMGL	0207	3OXS	FLPSDFFPSV	1.75 Å	452.77	-213.42	
P74	ILCWGELMNL	0207	3OXS	FLPSDFFPSV	1.75 Å	37.23	-206.901	
	ILCWGELMNL	2402	4F7M	LYASPQLEGF	2.4 Å	-6.42	-205.001	
P88	ASRELVVSY	0201	3MGT	KLYQNPTTYI	2.2 Å	9.63	-205.329	
	ASRELVVSY	3001	6J1W	AIFQSSMTK	1.5 Å	27.34	-192.458	
	ASRELVVSY	0206	3OXR	FLPSDFFPSV	1.7 Å	48.17	-188.493	
P118	ETVLEYLVSV	2601	6AT9(Template)	AQDIYRASYY	2.95 Å	76.53	-109.727	-90.805
	ETVLEYLVSV	1101	5GRD	SSCSSCPLSK	1.8Å	/	/	93.877
	ETVLEYLVSV	0201	3UTQ	ALWGPDPAAA	1.67 Å	-15.25	-211.162	-76.755
P96	ILSTLPETTV	0201	5YXU	KLVALGINAV	2.7 Å	18.05	-243.602	
	ILSTLPETTV	0203	3OX8	FLPSDFFPSV	2.16 Å	49.55	-192.699	
P111	LLDTASALY	0101	6AT9	AQDIYRASYY	2.95 Å	6.27	-222.514	
	LLDTASALY	1102	5WJL(Template)	GTSGSPIVNR	3.15Å	23.87	-205.293	
P16	PTYKAFLCK	1101	5WJN	GTSGSPIINR	2.85Å	8.94	-226.19	
	PTYKAFLCK	0206	3OXR	FLPSDFFPSV	1.7 Å	18.47	-218.295	
P25	FYPKVTKYL	2402	5HGH	RYPLTFGWCF	2.39 Å	5.55	-240.69	

	FYPKVTKYL	1101	1QVO	QVPLRPMTYK	2.22Å	115.94	-103.825	
P33	KLIGTHNSV (HID)	0201	1AO7	LLFGYPVYV	2.6 Å	66.41	-224.859	
	KLIGTHNSV (HID)	0203	3OX8	FLPSDFFPSV	2.16 Å	47.29	-194.958	
	KLIGTHNSV (HID)	1101	1X7Q	KTFPPTEPK	1.45Å	39.57	-190.801	
	KLIGTHNSV (HIP)	0203	3OX8	FLPSDFFPSV	2.16 Å	35.56	-206.683	
	KLIGTHNSV (HIP)	1101	1X7Q	KTFPPTEPK	1.45Å	30.02	-200.345	
	KLIGTHNSV (HIP)	0201	1QEW	FLWGPRALV	2.2 Å	26	-194.847	
	KLIGTHNSV (HIE)	0203	3OX8	FLPSDFFPSV	2.16 Å	37.2	-205.05	
	KLIGTHNSV (HIE)	1101	1X7Q	KTFPPTEPK	1.45Å	30.16	-200.209	
	KLIGTHNSV (HIE)	0201	1QEW	FLWGPRALV	2.2 Å	31.3	-189.542	
P34	KLIGTDNSV	0201	1AO7	LLFGYPVYV	2.6 Å	68.06	-223.205	
	KLIGTDNSV	0203	3OX8	FLPSDFFPSV	2.16 Å	55.46	-186.788	
P55	LYSSTVPCF	2402	3QZW	RYPLTFGWCF	2.8 Å	30.13	-224.354	
	LYSSTVPCF	3303	5WJL(Template)	GTSGSPIVNR	3.15 Å	23.65	-201.253	-62.735
	LYSSTVPCF	0201	1AO7	LLFGYPVYV	2.6 Å	95.69	-195.528	
P56	LYSSTVPVF	2402	5HGH	RYPLTFGWCF	2.39 Å	32.76	-215.287	
	LYSSTVPVF	0207	3OXS	FLPSDFFPSV	1.75 Å	55.55	-182.984	
	LYSSTVPVF	0201	4WUU	RMFPNAPYL	3.05 Å	83.19	-180.441	
P76	FLLAQFTSA	0203	3OX8	FLPSDFFPSV	2.16 Å	14.71	-224.393	
	FLLAQFTSA	0207	3OXS	FLPSDFFPSV	1.75 Å	18.52	-221.367	
P89	RSRSGAKLI	0201	4WUU	RMFPNAPYL	3.05 Å	29.69	-236.247	
	RSRSGAKLI	0206	3OXR	FLPSDFFPSV	1.7 Å	25.52	-212.082	
	RSRSGAKLI	3001	6J1W	AIFQSSMTK	1.5 Å	29.76	-190.118	
P90	RSRSGANIL	0201	4WUU	RMFPNAPYL	3.05 Å	28.17	-236.333	
	RSRSGANIL	3001	6J1W	AIFQSSMTK	1.5 Å	20.4	-199.485	
P98	LLAQFTSAI	0203	3OX8	FLPSDFFPSV	2.16 Å	47.89	-193.325	
P106	TLWKAGILYK	0301	3RL2	QVPLRPMTYK	2.39 Å	21.95	-198.361	
	TLWKAGILYK	2402	3QZW	RYPLTFGWCF	2.8 Å	64.92	-190.629	
	TLWKAGILYK	1102	5WJL(Template)	GTSGSPIVNR	3.15Å	49.1	-172.168	
P119	HTAELLAACF (HIP)	2402	4F7M	LYASPQLEGF	2.4 Å	18.23	-190.796	
	HTAELLAACF (HIP)	2601	6AT9(Template)	AQDIYRASYY	2.95 Å	61.74	-124.521	
	HTAELLAACF (HID)	2402	4F7M	LYASPQLEGF	2.4 Å	18.91	-190.117	
	HTAELLAACF (HID)	2601	6AT9(Template)	AQDIYRASYY	2.95 Å	69.62	-116.639	
	HTAELLAACF (HIE)	2402	4F7M	LYASPQLEGF	2.4 Å	20.01	-189.014	

	HTAELLAACF (HIE)	2601	6AT9(Template)	AQDIYRASYY	2.95 Å	56.89	-129.266	
P17	TVNAHQILPK	1101	1QVO	QVPLRPMTYK	2.22Å	/	/	-91.657
	TVNAHQILPK	3001	6JIW	AIFQSSMTK	1.5Å	/	/	-86.233
P18	STTDLEAYFK	0201	1I4F	GVYDGREHTV	1.4 Å	10.56	-190.192	
	STTDLEAYFK	1101	2HN7	AIMPARFYPK	1.6Å	30.65	-178.581	
	STTDLEAYFK	0206	3OXR	FLPSDFFPSV	1.7 Å	85.35	-151.319	
P26	VCAPAPCNF	2402	4F7M	LYASPQLEGF	2.4 Å	13.41	-195.616	
P47	TVNAHGNLPK (HIP)	0201	5YXN	KLVALGINAV	2.03 Å	18.4	-244.049	
	TVNAHGNLPK (HIP)	1101	2HN7	AIMPARFYPK	1.6Å	23.84	-185.088	
	TVNAHGNLPK (HIP)	0301	3RL2	QVPLRPMTYK	2.39 Å	53.71	-164.219	
	TVNAHGNLPK (HIE)	0201	5YXU	KLVALGINAV	2.7 Å	17.58	-236.718	
	TVNAHGNLPK (HIE)	1101	1QVO	QVPLRPMTYK	2.22Å	36.96	-180.271	
	TVNAHGNLPK (HIE)	0301	3RL2	QVPLRPMTYK	2.39 Å	60.05	-157.886	
	TVNAHGNLPK (HID)	0201	5YXN	KLVALGINAV	2.03 Å	31.83	-230.622	
	TVNAHGNLPK (HID)	1101	1QVO	QVPLRPMTYK	2.22Å	33.04	-184.185	
	TVNAHGNLPK (HID)	0301	3RL2	QVPLRPMTYK	2.39 Å	59.57	-158.362	
P48	TVNAHQVLPK (HID)	1101	1QVO	QVPLRPMTYK	2.22Å	23.27	-193.959	
	TVNAHQVLPK (HIE)	1101	1QVO	QVPLRPMTYK	2.22Å	24.38	-192.848	
	TVNAHQVLPK (HIP)	1101	1QVO	QVPLRPMTYK	2.22Å	25.12	-192.111	
P63	KVFVLGGCR	2402	5HGH	RYPLTFGWCF	2.39 Å	37.2	-208.423	
	KVFVLGGCR	3101	5WJL(Template)	GTSGSPIVNR	3.15Å	36.61	-189.256	
P93	RLKVFVLGG	2402	4F7M	LYASPQLEGF	2.4 Å	-16.37	-214.286	
	RLKVFVLGG	3001	6J1W	AIFQSSMTK	1.5 Å	24.83	-195.052	
P14	ASPLSSIFSR	1101	5WJN	GTSGSPIINR	2.85Å	16.55	-219.968	
P15	KVTKYLPLDK	1101	1QVO	QVPLRPMTYK	2.22Å	31.96	-185.267	
P13	SMYPSCCCTK	0201	1I4F	GVYDGREHTV	1.4 Å	-2.19	-203.585	-80.997
	SMYPSCCCTK	1101	2HN7	AIMPARFYPK	1.6Å	18.59	-194.147	-64.234
	SMYPSCCCTK	0301	3RL2	QVPLRPMTYK	2.39 Å	29.52	-188.695	76.071

Note: **Bond Peptide**: The original bond peptide in HLA structure. **Template**: The structure used as template in HLA homologous modelling task. **ΔAffinity**: Change in binding affinity of the specified binding partners. A negative value means that the mutant binds better than the original protein. **Prime Affinity**: The binding affinity between two sets of protein chains. And it was calculated with MM-GBSA in implicit solvent. **Binding Free Energy**: Due to the amino acid

properties of some HBV peptides, we firstly used peptide docking functionality in Biologics and molecular dynamics simulations in Desmond package(Schrodinger 2020-4 release) to get reasonable binding coformation of HLA molecules and HBV peptides. And then, we used MM-GBSA method calculated the binding free energy. The binding free energy represents the strength of the bond from the perspective of the total energy. In general, more negative the prime affinity, stronger the bond, and greater the energy is required to break a bond. A positive value indicates that bonding cannot occur spontaneously.

Figure S1:

```
                         10        20        30        40        50        60        70        80
                 ....|....|....|....|....|....|....|....|....|....|....|....|....|....|....|....|
HBsAg-C(P31868)  MGGWSSKPRQGMGTNLSVPNPLGFFPDHQLDPAFGANSNNPDWDFNPNKDHWPEANQVGVGTFGPGFTPPHGGLLGWSPQ
HBsAg-A(P31873)  .....A...K...................................................A................S.
HBsAg-B(Q67926)  .........K........................K...E.....L..H..N..D..K....A..................
HBsAg-D(P03139)  -----------..Q...TS...............R..T............T..D..K..A.A..L...............

                         90       100       110       120       130       140       150       160
                 ....|....|....|....|....|....|....|....|....|....|....|....|....|....|....|....|
HBsAg-C(P31868)  AQGILTTVPAAPPPASTNRQSGRQPTPISPPLRDSHPQAMQWNSTTFHQALLDPRVRGLYFPAGGSSSGTVNPVPTTASP
HBsAg-A(P31873)  ...T.H....V.........T........................A.Q...Q.......F.............A.NI..H
HBsAg-B(Q67926)  ...L.......................F......T............L.T.Q.S...A..L..........S.AQN.V.A
HBsAg-D(P03139)  ....MQ.L..N................L.....TT.....H........T.Q.........................T..

                        170       180       190       200       210       220       230       240
                 ....|....|....|....|....|....|....|....|....|....|....|....|....|....|....|....|
HBsAg-C(P31868)  ISSIFSRTGDPAPNMENTTSGFLGPLLVLQAGFFLLTRILTIPQSLDSWWTSLNFLGGAPTCPGQNSQSPTSNHSPTSCP
HBsAg-A(P31873)  ....S.......L....I........................................S.V.L.................
HBsAg-B(Q67926)  ....S.K....V.....IA..L..H.........S..K....................T.A........QI.S....C..
HBsAg-D(P03139)  .......I....L....I........................................TTV.L.......I.........

                        250       260       270       280       290       300       310       320
                 ....|....|....|....|....|....|....|....|....|....|....|....|....|....|....|....|
HBsAg-C(P31868)  PICPGYRWMCLRRFIIFLFILLLCLIFLLVLLDYQGMLPVCPLLPGTSTTSTGPCKTCTTPAQGTSMFPSCCCTKPSDGN
HBsAg-A(P31873)  ...........................................I..ST................N...........T...
HBsAg-B(Q67926)  ..................C........................T..ST............................T...
HBsAg-D(P03139)  .T.........................................I..S......S.R........I..Y............

                        330       340       350       360       370       380       390       400
                 ....|....|....|....|....|....|....|....|....|....|....|....|....|....|....|....|
HBsAg-C(P31868)  CTCIPIPSSWAFARFLWEWASVRFSWLSLLVPFVQWFAGLSPTVWLSVIWMMWYWGPSLYNILSPFLPLLPIFFCLWVYI
HBsAg-A(P31873)  .............KY......................V............................I.............
HBsAg-B(Q67926)  .............KY..G...................V...............F.........R..M....T........
HBsAg-D(P03139)  ............GK.......A...............V....I.................S................A..


                         10        20        30        40        50        60        70        80
                 ....|....|....|....|....|....|....|....|....|....|....|....|....|....|....|....|
HBeAg-C(P0C6H5)  MQLFHLCLIISCSCPTVQASKLCLGWLWGMDIDPYKEFGASVELLSFLPSDFFPSIRDLLDTASALYREALESPEHCSPH
HBeAg-A(Q91C37)  ............T...........................T..............V........................
HBeAg-B(P0C6G7)  ................................................................................
HBeAg-D(P0C573)  ........................................T..............V........................

                         90       100       110       120       130       140       150       160
                 ....|....|....|....|....|....|....|....|....|....|....|....|....|....|....|....|
HBeAg-C(P0C6H5)  HTALRQAILCWGELMNLATWVGSNLEDPASRELVVSYVNVNMGLKIRQLLWFHISCLTFGRETVLEYLVSFGVWIRTPPA
HBeAg-A(Q91C37)  .....ET............T......N........D...N...T........................................
HBeAg-B(P0C6G7)  ................................................................................
HBeAg-D(P0C573)  ...............T......V........D.......T.....F..................I...............

                        170       180       190       200       210
                 ....|....|....|....|....|....|....|....|....|....|....
HBeAg-C(P0C6H5)  YRPPNAPILSTLPETTVVRRRGRSPRRRTPSPRRRRSQSPRRRRSQSRESQC
HBeAg-A(Q91C37)  .....................D.GRSP.RRT.SP..RR.QSP..RR.QSRESQC
HBeAg-B(P0C6G7)  ....................................................
HBeAg-D(P0C573)  ....................................................


                         10        20        30        40        50        60        70        80
                 ....|....|....|....|....|....|....|....|....|....|....|....|....|....|....|....|
HBx-C(P0C686)    MAARVCCQLDPARDVLCLRPVGAESRGRPVSGPFGPLPSPSSSAVPADHGARLSLRGLPVCAFSSAGPCALRFTSARRME
HBx-A(Q91C38)    ....LY....SS.................LA..L.A.S...P....S....H.........................C..
HBx-B(Q67923)    .............................LP..L.T..PA.PP...T....H............................
HBx-D(Q9QMI3)    ....L............................L.S.S.S.P....T....H............................

                         90       100       110       120       130       140       150
                 ....|....|....|....|....|....|....|....|....|....|....|....|....|....|....
HBx-C(P0C686)    TTVNAHQVLPKVLHKRTLGLSAMSTTDLEAYFKDCLFKDWEELGEEIRLMVFVLGGCRHKLVCSPAPCNFFTSA
HBx-A(Q91C38)    .......I............P..............V.............K............FA.SS.......
HBx-B(Q67923)    ......GN...........................V.NE..........K........................
HBx-D(Q9QMI3)    .......I...I.........T...........................K.............A..........
```

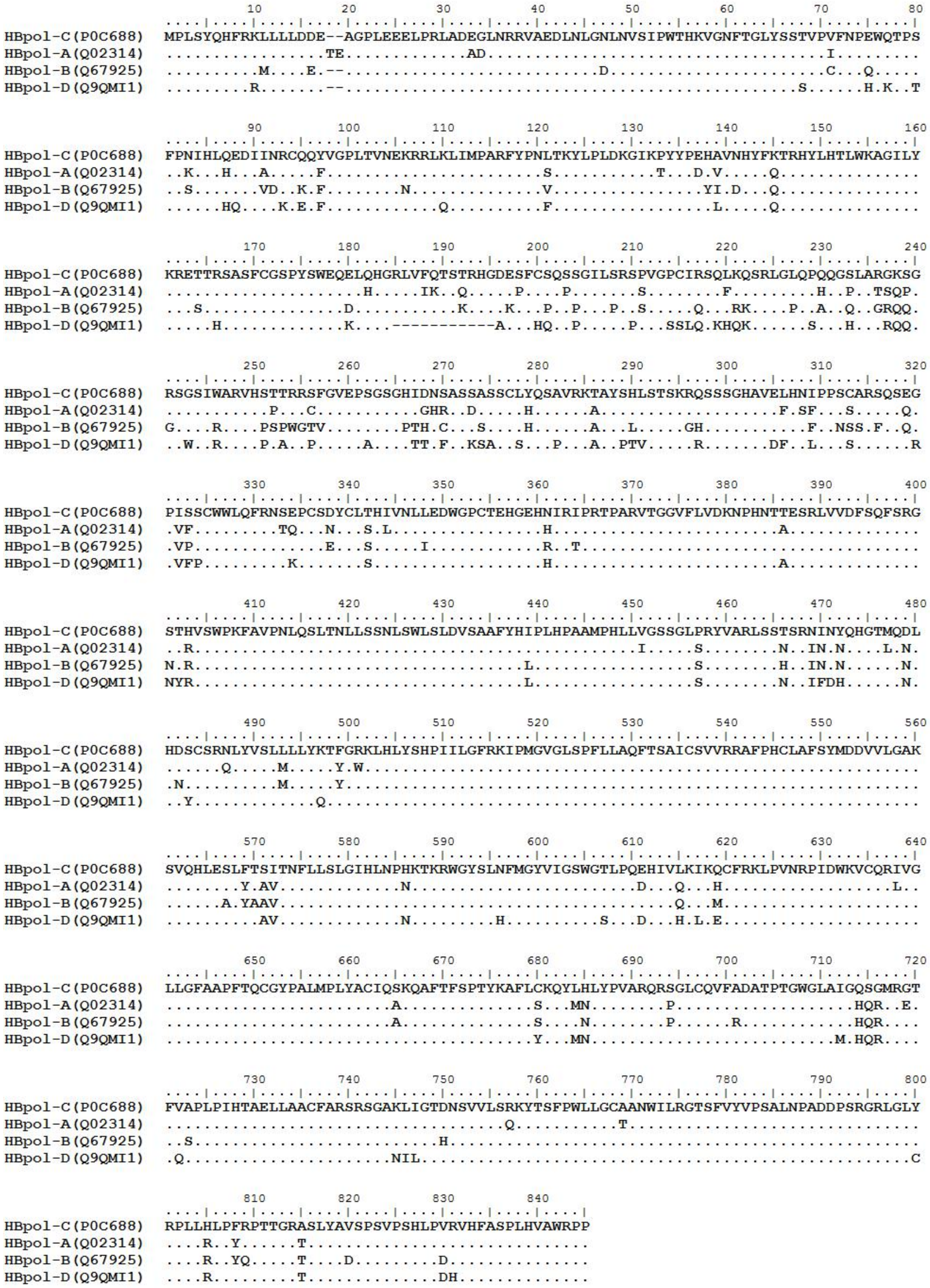

Figure S1: Homologous analysis of HBsAg, HBeAg, HBx and HBpol proteins from HBV C, A, B, and D genotypes. The entire amino acid sequences of each protein from different genotypes of HBV were obtained from UniProt database, aligned and used for *in silico* prediction of HBV antigen T cell epitopes presented by HLA-A allotyps.

Figure S2:

A

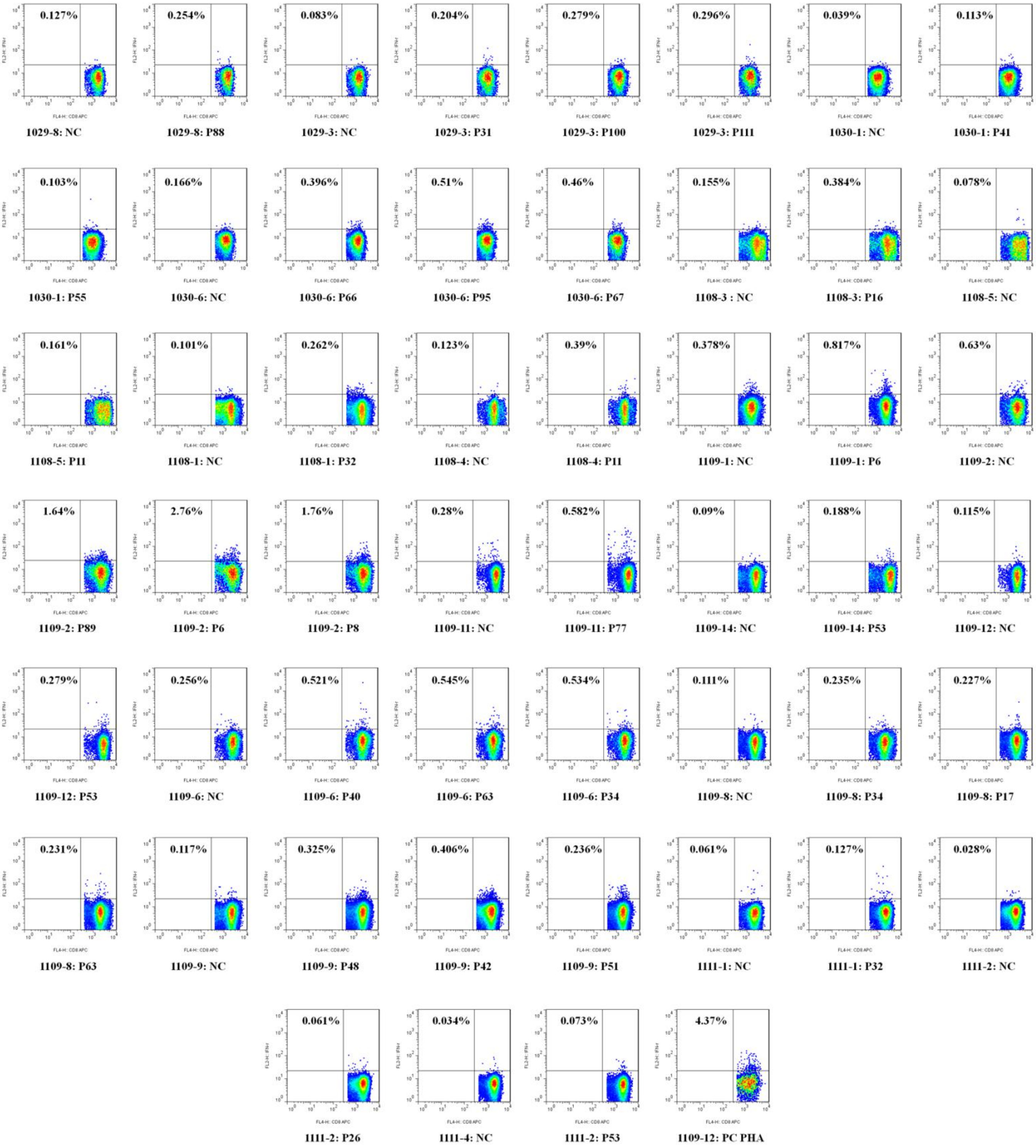
0.127%
1029-8: NC
0.254%
1029-8: P88
0.083%
1029-3: NC
0.204%
1029-3: P31
0.279%
1029-3: P100
0.296%
1029-3: P111
0.039%
1030-1: NC
0.113%
1030-1: P41
0.103%
1030-1: P55
0.166%
1030-6: NC
0.396%
1030-6: P66
0.51%
1030-6: P95
0.46%
1030-6: P67
0.155%
1108-3 : NC
0.384%
1108-3: P16
0.078%
1108-5: NC
0.161%
1108-5: P11
0.101%
1108-1: NC
0.262%
1108-1: P32
0.123%
1108-4: NC
0.39%
1108-4: P11
0.378%
1109-1: NC
0.817%
1109-1: P6
0.63%
1109-2: NC
1.64%
1109-2: P89
2.76%
1109-2: P6
1.76%
1109-2: P8
0.28%
1109-11: NC
0.582%
1109-11: P77
0.09%
1109-14: NC
0.188%
1109-14: P53
0.115%
1109-12: NC
0.279%
1109-12: P53
0.256%
1109-6: NC
0.521%
1109-6: P40
0.545%
1109-6: P63
0.534%
1109-6: P34
0.111%
1109-8: NC
0.235%
1109-8: P34
0.227%
1109-8: P17
0.231%
1109-8: P63
0.117%
1109-9: NC
0.325%
1109-9: P48
0.406%
1109-9: P42
0.236%
1109-9: P51
0.061%
1111-1: NC
0.127%
1111-1: P32
0.028%
1111-2: NC
0.061%
1111-2: P26
0.034%
1111-4: NC
0.073%
1111-2: P53
4.37%
1109-12: PC PHA

B:

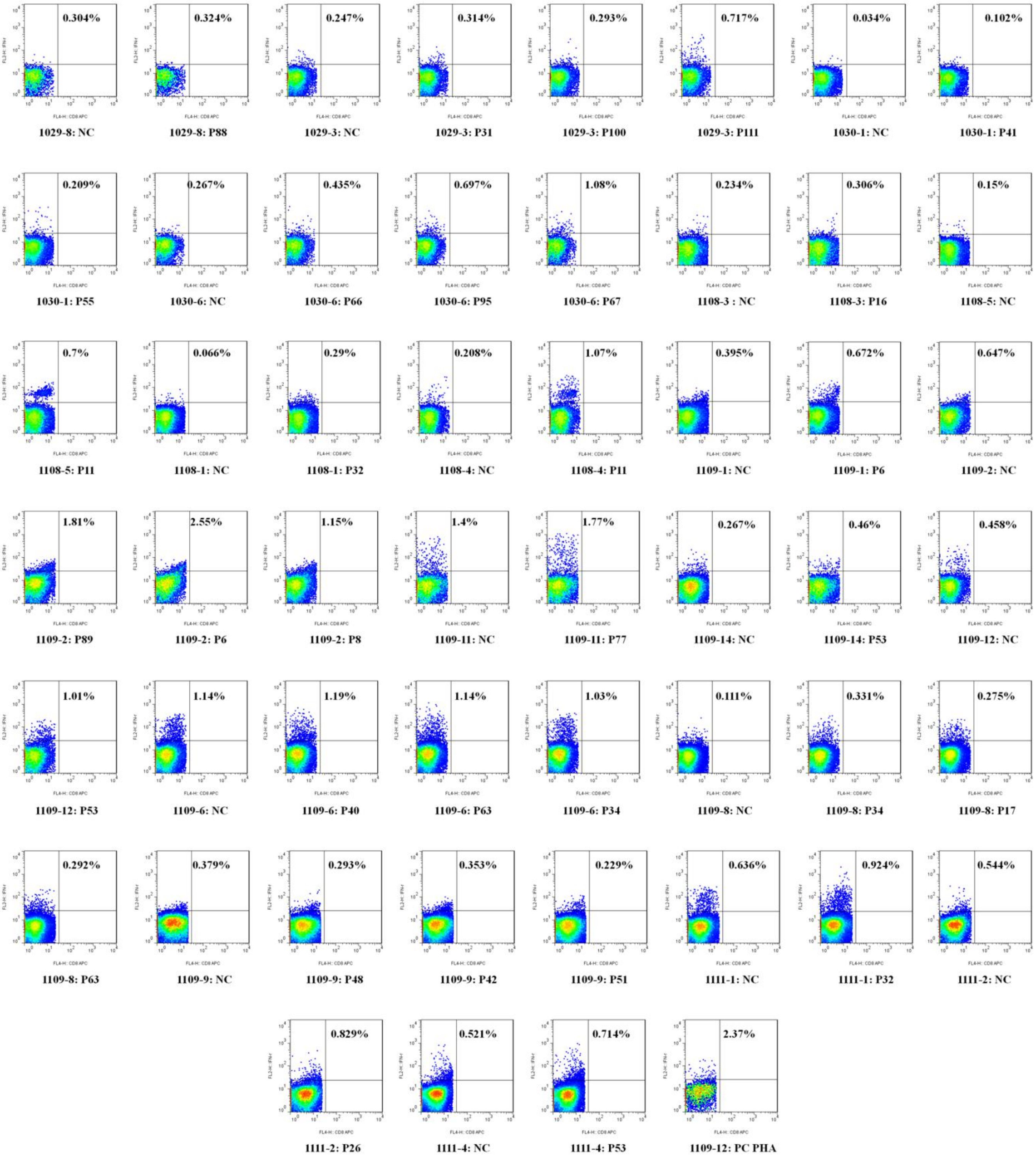

Figure S2: The validated HBV epitope peptides induced CD8^{+} T cell responses in the *in vitro* cocultures with patients' PBMCs. The PBMCs were stimulated with the indicated epitope peptides for seven days *in vitro* and followed by IFN-γ intracellular staining. The frequencies of IFN-γ^{+} cells in CD3^{+}/CD8^{+} or CD3^{+}/CD8^{-} populations were analyzed by flow

cytometry. Totally, 25 epitope peptides were tested using the PBMCs from 19 patients with matching HLA-A alleles. (A) The flow plots of IFN-γ^+ cells in $CD3^+/CD8^+$ T cell population in each coculture. (B) The flow plots of IFN-γ^+ cells in $CD3^+/CD8^-$ T cell population in each coculture.

Figure S3:

HBV C genotype-HBsAg

sp|P31868|:

MGGWSSKPRQGMGTNLSVPNPLGFFPDHQLDPAFGANSNNPDWDFNPNKDHWPEANQVGVGT
FGPGFTPPHGGLLGWSPQAQGILTTVPAAPPPASTNRQSGRQPTPISPPLRDSHPQAMQWNS
TTFHQALLDPRVRGLYFPAGGSSSGTVNPVPTTASPISSIFSRTGDPAPNMENTTSGFLGPL
LVLQAGFFLLTRILTIPQSLDSWWTSLNFLGGAPTCPGQNSQSPTSNHSPTSCPPICPGYRW
MCLRRFIIFLFILLLCLIFLLVLLDYQGMLPVCPLLPGTSTTSTGPCKTCTTPAQGTSMFPS
CCCTKPSDGNCTCIPIPSSWAFARFLWEWASVRFSWLSLLVPFVQWFAGLSPTVWLSVIWMM
WYWGPSLYNILSPFLPLLPIFFCLWVYI

HBV C genotype-HBeAg

sp|P0C6H5|:

SKLCLGWLWGMDIDPYKEFGASVELLSFLPSDFFPSIRDLLDTASALYREALESPEHCSPHH
TALRQAILCWGELMNLATWVGSNLEDPASRELVVSYVNVNMGLKIRQLLWFHISCLTFGRET
VLEYLVSFGVW

HBV C genotype-HBx

sp|P0C686|:

MAARVCCQLDPARDVLCLRPVGAESRGRPVSGPFGPLPSPSSSAVPADHGARLSLRGLPVCA
FSSAGPCALRFTSARRMETTVNAHQVLPKVLHKRTLGLSAMSTTDLEAYFKDCLFKDWEELG
EEIRLMVFVLGGCRHKLVCSPAPCNFFTSA

HBV C genotype-HBpol

sp|P0C688|:

MPLSYQHFRKLLLLDDEAGPLEEELPRLADEGLNRRVAEDLNLGNLNVSIPWTHKVGNFTGL
YSSTVPVFNPEWQTPSFPNIHLQEDIINRCQQYVGPLTVNEKRRLKLIMPARFYPNLTKYLP
LDKGIKPYYPEHAVNHYFKTRHYLHTLWKAGILYKRETTRSASFCGSPYSWEQELQHGRLVF
QTSTRHGDESFCSQSSGILSRSPVGPCIRSQLKQSRLGLQPQQGSLARGKSGRSGSIWARVH
STTRRSFGVEPSGSGHIDNSASSASSCLYQSAVRKTAYSHLSTSKRQSSSGHAVELHNIPPS
CARSQSEGPISSCWWLQFRNSEPCSDYCLTHIVNLLEDWGPCTEHGEHNIRIPRTPARVTGG
VFLVDKNPHNTTESRLVVDFSQFSRGSTHVSWPKFAVPNLQSLTNLLSSNLSWLSLDVSAAF

YHIPLHPAAMPHLLVGSSGLPRYVARLSSTSRNINYQHGTMQDLHDSCSRNLYVSLLLLYKT
FGRKLHLYSHPIILGFRKIPMGVGLSPFLLAQFTSAICSVVRRAFPHCLAFSYMDDVVLGAK
SVQHLESLFTSITNFLLSLGIHLNPHKTKRWGYSLNFMGYVIGSWGTLPQEHIVLKIKQCFR
KLPVNRPIDWKVCQRIVGLLGFAAPFTQCGYPALMPLYACIQSKQAFTFSPTYKAFLCKQYL
HLYPVARQRSGLCQVFADATPTGWGLAIGQSGMRGTFVAPLPIHTAELLAACFARSRSGAKL
IGTDNSVVLSRKYTSFPWLLGCAANWILRGTSFVYVPSALNPADDPSRGRLGLYRPLLHLPF
RPTTGRASLYAVSPSVPSHLPVRVHFASPLHVAWRPP

HBV B genotype-HBsAg

sp|Q67926|

MGGWSSKPRKGMGTNLSVPNPLGFFPDHQLDPAFKANSENPDWDLNPHKDNWPDANKVGVGA
FGPGFTPPHGGLLGWSPQAQGLLTTVPAAPPPASTNRQSGRQPTPFSPPLRDTHPQAMQWNS
TTFLQTLQDSRVRALYLPAGGSSSGTVSPAQNTVSAISSISSKTGDPVPNMENIASGLLGHL
LVLQAGFFSLTKILTIPQSLDSWWTSLNFLGGTPACPGQNSQSQISSHSPTCCPPICPGYRW
MCLRRFIIFLCILLLCLIFLLVLLDYQGMLPVCPLTPGSTTTSTGPCKTCTTPAQGTSMFPS
CCCTKPTDGNCTCIPIPSSWAFAKYLWGWASVRFSWLSLLVPFVQWFVGLSPTVWLSVIWMM
WFWGPSLYNILRPFMPLLPTFFCLWVYI

HBV B genotype-HBeAg

sp|P0C6G7|

SKLCLGWLWGMDIDPYKEFGASVELLSFLPSDFFPSIRDLLDTASALYREALESPEHCSPHH
TALRQAILCWGELMNLATWVGSNLEDPASRELVVSYVNVNMGLKIRQLLWFHISCLTFGRET
VLEYLVSFGVW

HBV B genotype-HBx

sp|Q67923|

MAARVCCQLDPARDVLCLRPVGAESRGRPLPGPLGTLPPASPPAVPTDHGAHLSLRGLPVCA
FSSAGPCALRFTSARRMETTVNAHGNLPKVLHKRTLGLSAMSTTDLEAYFKDCVFNEWEELG
EEIRLKVF VLGGCRHKLVCSPAPCNFFTSA

HBV B genotype-HBpol

sp|Q67925| :

MPLSYQHFRKMLLLDEEAGPLEEELPRLADEGLNRRVAEDLNLGDLNVSIPWTHKVGNFTGL
YSSTVPCFNPQWQTPSFPSIHLQEDIVDRCKQFVGPLTVNENRRLKLIMPARFYPNVTKYLP
LDKGIKPYYPEYIVDHYFQTRHYLHTLWKAGILYKRESTRSASFCGSPYSWEQDLQHGRLVF
QTSKRHGDKSFCPQSPGILPRSSVGPCIQSQLRKSRLGPQPAQGQLAGRQQGGSGSIRARVH
PSPWGTVGVEPSGSGTHNCASSSSSCLHQSAVRKAAYSLLSTSKGHSSSGHAVELHNFPPNS
SRFQSQGPVPSCWWLQFRNSEPCSEYCLSHIVNLIEDWGPCTEHGEHRIRTPRTPARVTGGV
FLVDKNPHNTTESRLVVDFSQFSRGNTRVSWPKFAVPNLQSLTNLLSSNLSWLSLDVSAAFY
HLPLHPAAMPHLLVGSSGLSRYVARLSSHSRINNNQHGTMQNLHNSCSRNLYVSLMLLYKTY

GRKLHLYSHPIILGFRKIPMGVGLSPFLLAQFTSAICSVVRRAFPHCLAFSYMDDVVLGAKS
VQHLEALYAAVTNFLLSLGIHLNPHKTKRWGYSLNFMGYVIGSWGTLPQEHIVQKIKMCFRK
LPVNRPIDWKVCQRIVGLLGFAAPFTQCGYPALMPLYACIQAKQAFTFSPTYKAFLSKQYLN
LYPVARQRPGLCQVFRDATPTGWGLAIGHQRMRGTFVSPLPIHTAELLAACFARSRSGAKLI
GTHNSVVLSRKYTSFPWLLGCAANWILRGTSFVYVPSALNPADDPSRGRLGLYRPLLRLPYQ
PTTGRTSLYADSPSVPSHLPDRVHFASPLHVAWRPP

HBV A genotype-HBsAg

sp|P31873| :

MGGWSAKPRKGMGTNLSVPNPLGFFPDHQLDPAFGANSNNPDWDFNPNKDHWPEANQVGVGA
FGPGFTPPHGGLLGWSSQAQGTLHTVPAVPPPASTNRQTGRQPTPISPPLRDSHPQAMQWNS
TAFQQALQDPRVRGLFFPAGGSSSGTVNPAPNIASHISSISSRTGDPALNMENITSGFLGPL
LVLQAGFFLLTRILTIPQSLDSWWTSLNFLGGSPVCLGQNSQSPTSNHSPTSCPPICPGYRW
MCLRRFIIFLFILLLCLIFLLVLLDYQGMLPVCPLIPGSTTTSTGPCKTCTTPAQGNSMFPS
CCCTKPTDGNCTCIPIPSSWAFAKYLWEWASVRFSWLSLLVPFVQWFVGLSPTVWLSVIWMM
WYWGPSLYNILSPFIPLLPIFFCLWVYI

HBV A genotype-HBeAg

sp|Q91C37| :

SKLCLGWLWGMDIDPYKEFGATVELLSFLPSDFFPSVRDLLDTASALYREALESPEHCSPHH
TALRETILCWGELMTLATWVGNNLEDPASRDLVVNYVNTNMGLKIRQLLWFHISCLTFGRET
VLEYLVSFGVW

HBV A genotype-HBx

sp|Q91C38| :

MAARLYCQLDSSRDVLCLRPVGAESRGRPLAGPLGALSSPSPSAVPSDHGAHLSLRGLPVCA
FSSAGPCALRFTSARCMETTVNAHQILPKVLHKRTLGLPAMSTTDLEAYFKDCVFKDWEELG
EEIRLKVFVLGGCRHKLVFAPSSCNFFTSA

HBV A genotype-HBpol

sp|Q02314| :

MPLSYQHFRKLLLLDDETEAGPLEEELPRLADADLNRRVAEDLNLGNLNVSIPWTHKVGNFT
GLYSSTVPIFNPEWQTPSFPKIHLHEDIANRCQQFVGPLTVNEKRRLKLIMPARFYPNSTKY
LPLDKGIKTYYPDHVVNHYFQTRHYLHTLWKAGILYKRETTRSASFCGSPYSWEQELHHGRL
VIKTSQRHGDEPFCSQPSGILSRSSVGPCIRSQFKQSRLGLQPHQGPLATSQPGRSGSIWAR
VHSPTRRCFGVEPSGSGHIGHRASDASSCLHQSAVRKAAYSHLSTSKRQSSSGHAVEFHSFP
PSSARSQSQGPVFSCWWLQFRNTQPCSNYCLSHLVNLLEDWGPCTEHGEHHIRIPRTPARVT
GGVFLVDKNPHNTAESRLVVDFSQFSRGSTRVSWPKFAVPNLQSLTNLLSSNLSWLSLDVSA
AFYHIPLHPAAMPHLLIGSSGLSRYVARLSSNSRINNNQHGTLQNLHDSCSRQLYVSLMLLY
KTYGWKLHLYSHPIILGFRKIPMGVGLSPFLLAQFTSAICSVVRRAFPHCLAFSYMDDVVLG

AKSVQHLESLYTAVTNFLLSLGIHLNPNKTKRWGYSLNFMGYVIGSWGTLPQDHIVQKIKHC
FRKLPVNRPIDWKVCQRLVGLLGFAAPFTQCGYPALMPLYACIQAKQAFTFSPTYKAFLSKQ
YMNLYPVARQRPGLCQVFADATPTGWGLAIGHQRMRETFVAPLPIHTAELLAACFARSRSGA
KLIGTDNSVVLSQKYTSFPWLLGCTANWILRGTSFVYVPSALNPADDPSRGRLGLYRPLLRL
PYRPTTGRTSLYAVSPSVPSHLPVRVHFASPLHVAWRPP

HBV D genotype-HBsAg

sp|P03139| :

MGQNLSTSNPLGFFPDHQLDPAFRANTNNPDWDFNPNKDTWPDANKVGAGAFGLGFTPPHGG
LLGWSPQAQGIMQTLPANPPPASTNRQSGRQPTPLSPPLRTTHPQAMHWNSTTFHQTLQDPR
VRGLYFPAGGSSSGTVNPVPTTTSPISSIFSRIGDPALNMENITSGFLGPLLVLQAGFFLLT
RILTIPQSLDSWWTSLNFLGGTTVCLGQNSQSPISNHSPTSCPPTCPGYRWMCLRRFIIFLF
ILLLCLIFLLVLLDYQGMLPVCPLIPGSSTTSTGSCRTCTTPAQGISMYPSCCCTKPSDGNC
TCIPIPSSWAFGKFLWEWASARFSWLSLLVPFVQWFVGLSPIVWLSVIWMMWYWGPSLYSIL
SPFLPLLPIFFCLWAYI

HBV D genotype-HBeAg

sp|P0C573| :

SKLCLGWLWGMDIDPYKEFGATVELLSFLPSDFFPSVRDLLDTASALYREALESPEHCSPHH
TALRQAILCWGELMTLATWVGVNLEDPASRDLVVSYVNTNMGLKFRQLLWFHISCLTFGRET
VIEYLVSFGVW

HBV D genoype-HBx

sp|Q9QMI3| :

MAARLCCQLDPARDVLCLRPVGAESRGRPVSGPLGSLSSSSPSAVPTDHGAHLSLRGLPVCA
FSSAGPCALRFTSARRMETTVNAHQILPKILHKRTLGLSTMSTTDLEAYFKDCLFKDWEELG
EEIRLKVFVLGGCRHKLVCAPAPCNFFTSA

HBV D genotype-HBpol

sp|Q9QMI1|

MPLSYQHFRRLLLLDDEAGPLEEELPRLADEGLNRRVAEDLNLGNLNVSIPWTHKVGNFTGL
YSSSVPVFNPHWKTPTFPNIHLHQDIINKCEQFVGPLTVNEKRRLQLIMPARFYPNFTKYLP
LDKGIKPYYPEHLVNHYFQTRHYLHTLWKAGILYKRETTHSASFCGSPYSWEQKLQHGAESF
HQQSPGILSRPPVGSSLQSKHQKSRLGLQSQQGHLARRQQGRSWSIRARVHPTARRPFGVEP
AGSGHTTNFASKSASCSYQSPVRKAAYPTVSTSKRRSSSGHAVDFHNLPPSSARSQSERPVF
PCWWLQFRNSKPCSDYCLSHIVNLLEDWGPCTEHGEHHIRIPRTPARVTGGVFLVDKNPHNT
AESRLVVDFSQFSRGNYRVSWPKFAVPNLQSLTNLLSSNLSWLSLDVSAAFYHLPLHPAAMP
HLLVGSSGLSRYVARLSSNSRIFDHQHGTMQNLHDYCSRNLYVSLLLLYQTFGRKLHLYSHP
IILGFRKIPMGVGLSPFLLAQFTSAICSVVRRAFPHCLAFSYMDDVVLGAKSVQHLESLFTA
VTNFLLSLGIHLNPNKTKRWGYSLHFMGYVIGSWGSLPQDHIVHKLKECFRKLPVNRPIDWK

VCQRIVGLLGFAAPFTQCGYPALMPLYACIQSKQAFTFSPTYKAFLYKQYMNLYPVARQRSG
LCQVFADATPTGWGLAMGHQRMRGTFQAPLPIHTAELLAACFARSRSGANILGTDNSVVLSR
KYTSFPWLLGCAANWILRGTSFVYVPSALNPADDPSRGRLGLCRPLLRLPFRPTTGRTSLYA
VSPSVPSHLPDHVHFASPLHVAWRPP

Figure S3: Consensus sequences of HBsAg, HBeAg, HBx and HBpol in different HBV genotypes. A huge number of sequences of HBsAg, HBeAg, HBx and HBpol in HBV genotype A, B, C and D were collected from HBVdb database. Multiple sequence alignments were performed and analyzed to obtain the conservative regions of these sequences in a genotype-dependent way. The conservative properties of each amino acid in each protein was judged by the threshold of 100%, 95%, 80%, and highlighted the amino acids in different colors (100%: red, ≥95%: yellow, ≥80%: black in gray background, and <80%: black in white background).

Figure S4：

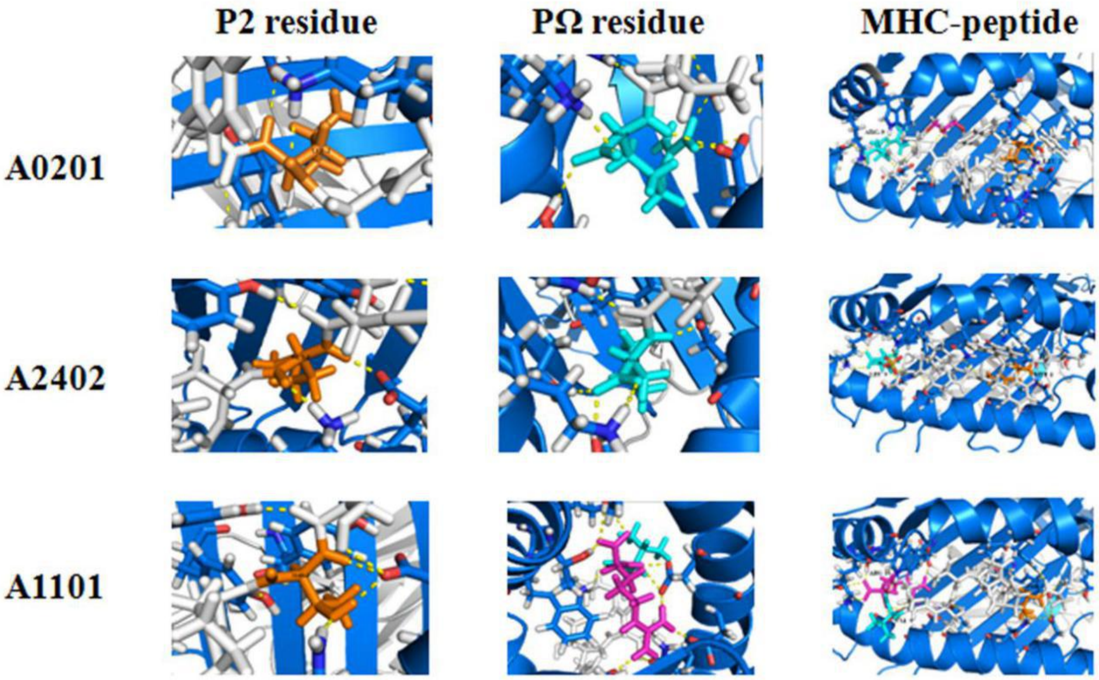

Figure S4: The binding pattern between the main anchor residues of HBV epitope peptides with HLA-A molecules. The conformational interaction between P2, PΩ and the whole length of HBV epitope peptide (FLWEWASVR), (MMWYWGPSL) and (STLPETTVVR) with HLA-A0201 (PDB ID: 5F9J), A2402 (PDB ID: 2BCK) and A1101 (PDB ID: 5WKF), respectively, were displayed in 3D structures of peptide-binding groove of HLA-A molecules.

Figure S5:

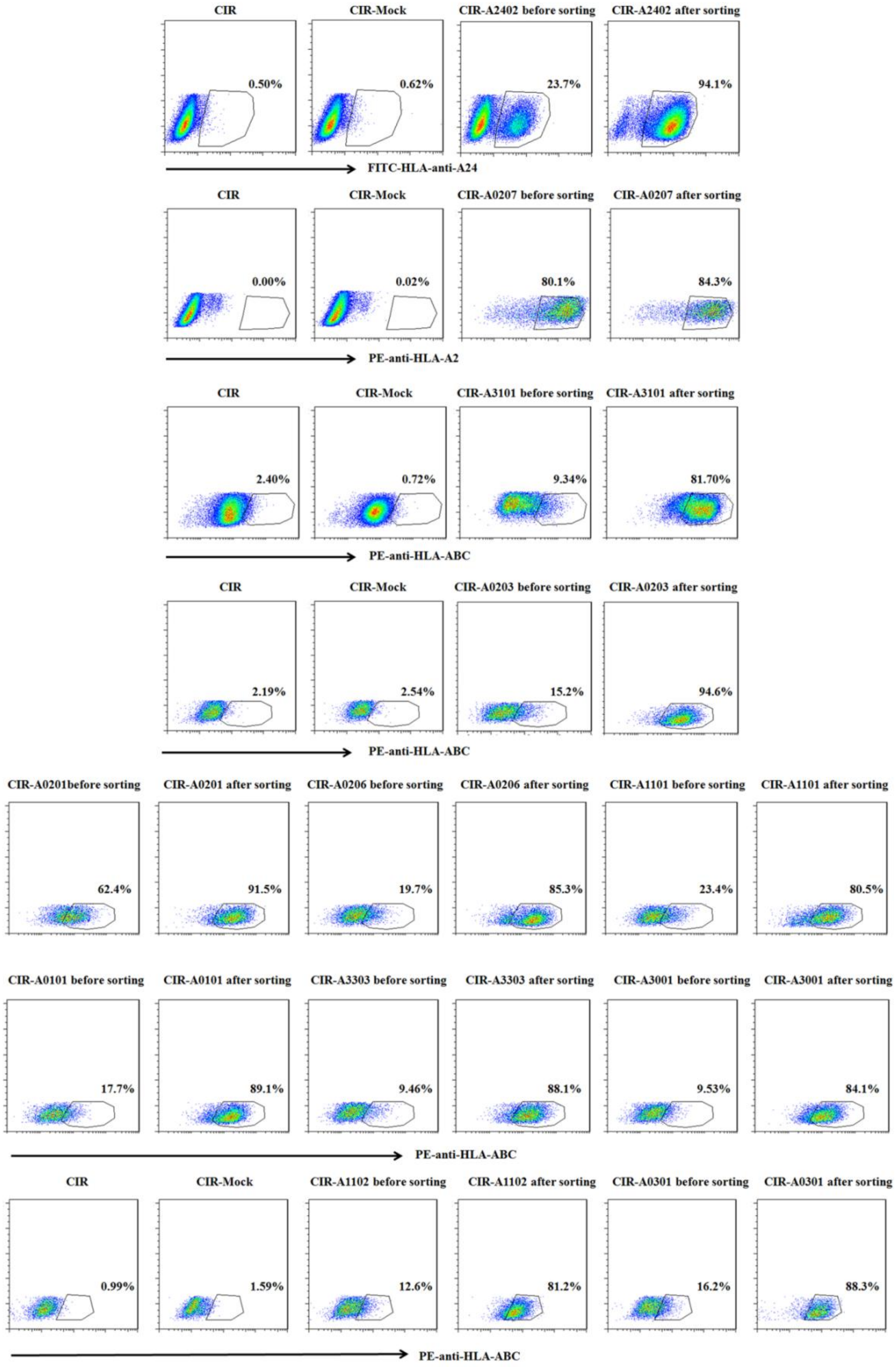

Figure S5: Hmy2.CIR cell lines expressing indicated HLA-A allotypes. The transfected Hmy2.1 CIR cell lines expressing HLA-A2402, A0207, A0201, A0203, A0206, A0101, A1101, A3101, A3303, A3001, A1102 or A0301 were generated, respectively, and stained

with FITC-anti-HLA-A24, PE-anti-HLA-A2 or PE-anti-HLA-ABC, then sorted by flow cytometry. The purity of these transfected HMy2.CIR cell lines was 80% to 94% after sorting.

Figure S6:

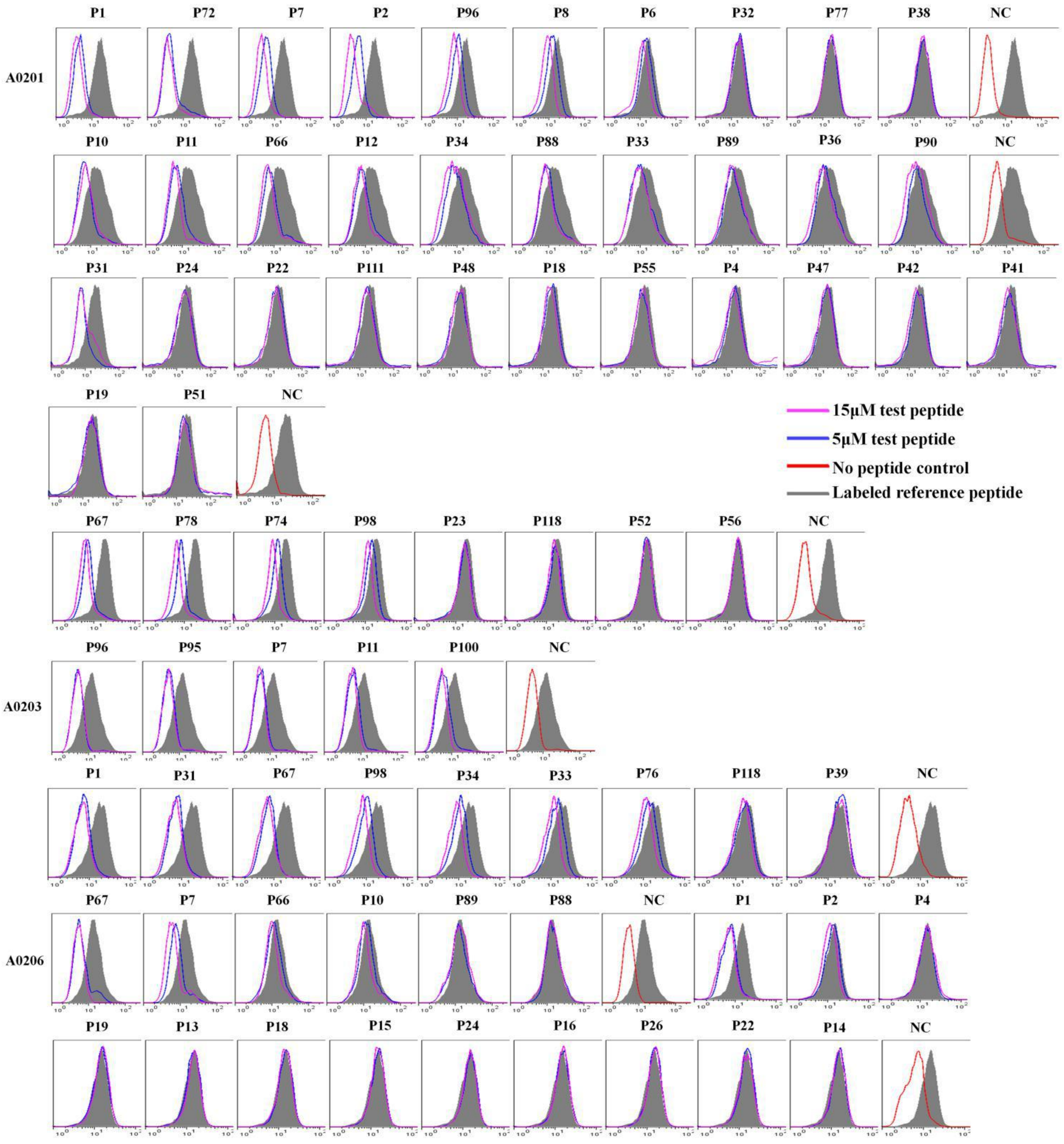

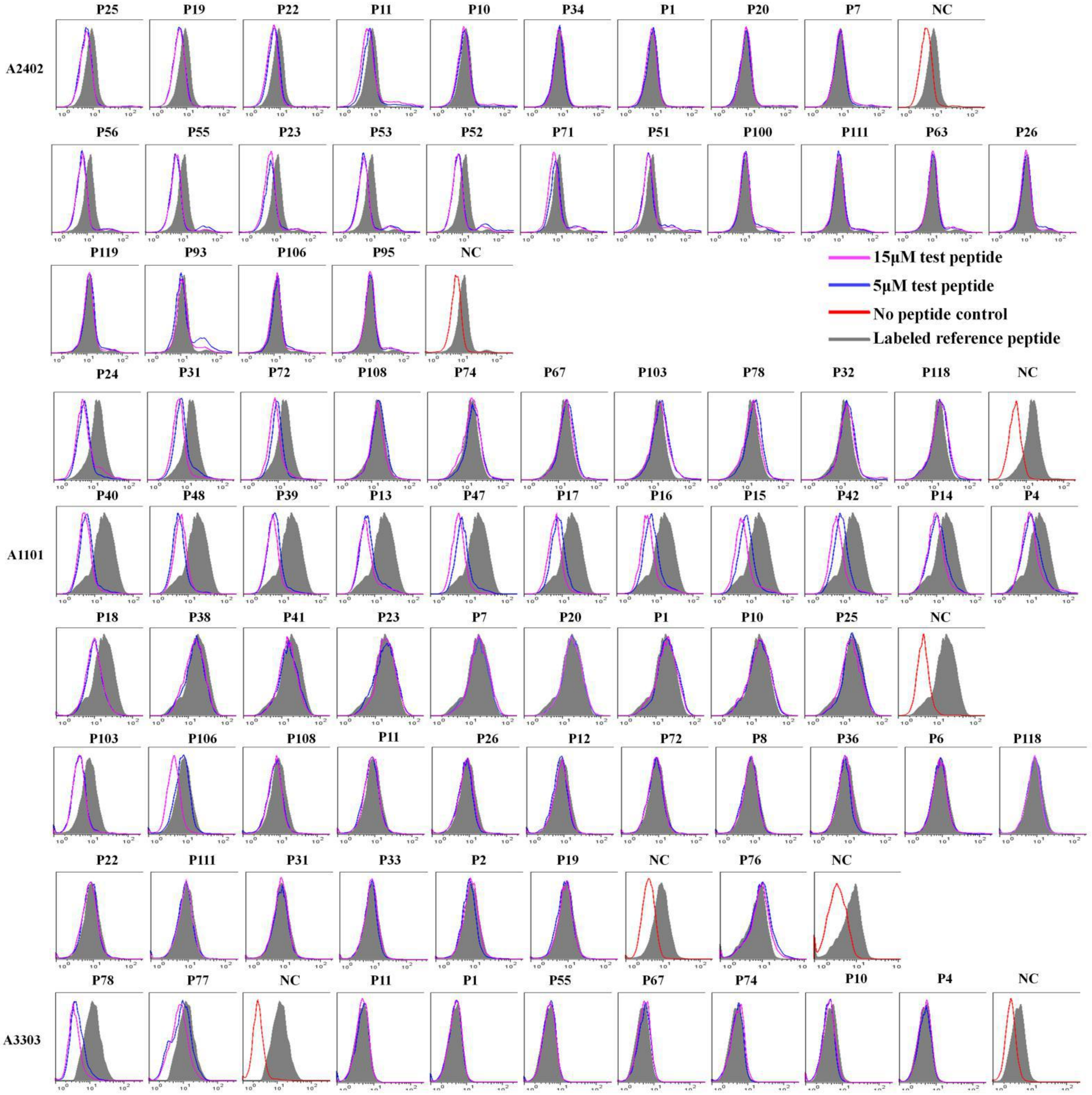
A2402
P25 P19 P22 P11 P10 P34 P1 P20 P7 NC
P56 P55 P23 P53 P52 P71 P51 P100 P111 P63 P26
P119 P93 P106 P95 NC
15μM test peptide
5μM test peptide
No peptide control
Labeled reference peptide
A1101
P24 P31 P72 P108 P74 P67 P103 P78 P32 P118 NC
P40 P48 P39 P13 P47 P17 P16 P15 P42 P14 P4
P18 P38 P41 P23 P7 P20 P1 P10 P25 NC
P103 P106 P108 P11 P26 P12 P72 P8 P36 P6 P118
P22 P111 P31 P33 P2 P19 NC P76 NC
A3303
P78 P77 NC P11 P1 P55 P67 P74 P10 P4 NC

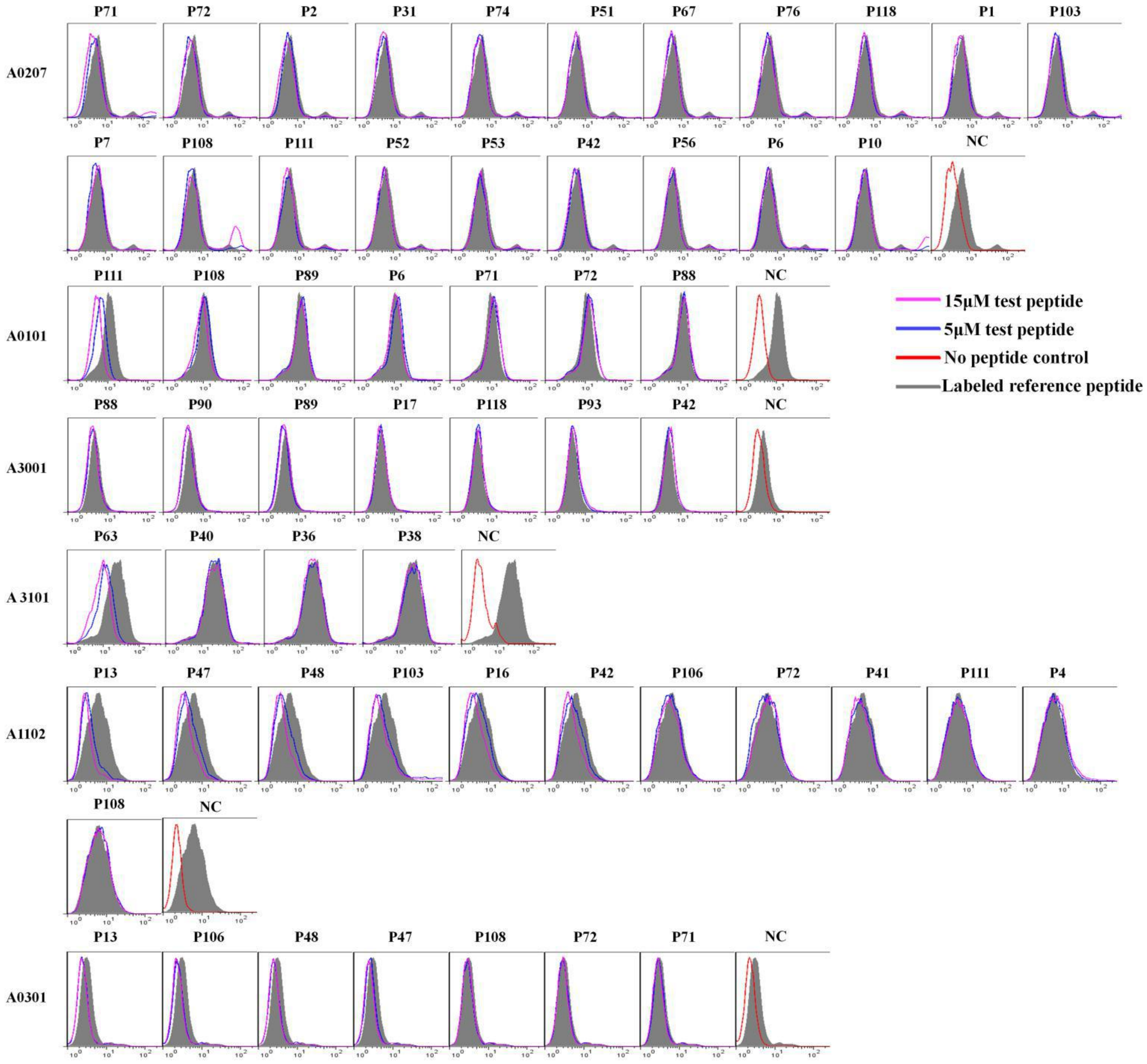

Figure S6: Binding affinity analysis of 62 validated HBV epitope peptides with corresponding HLA-A allotypes using peptide competitive binding assay. A series of unlabeled HBV epitope peptides were co-incubated with fluoresce-labeled reference peptides and transfected HMy2.CIR cell lines expressing the indicated HLA-A allotypes. Then the competitively binding (%) of each HBV epitope peptide at 5μM and 15μM was calculated by measuring the percentage of fluorescence-positive HMy2.CIR cells. Blue line and purple line represented the line diagram of cell fluorescence strength under 5μM and 15μM HBV epitope peptide, respectively. Gray filled and red line represented the cell maximal fluorescence (cells with FITC-labeled reference peptide) and negative control (cell background fluorescence with 1640 medium alone), respectively.
